# Piezo1 Activation Rescues Anabolic Response to Mechanical Loading in Aged Bone via Connexin 43 Hemichannels

**DOI:** 10.1111/acel.70687

**Published:** 2026-08-21

**Authors:** Dezhi Zhao, Jiayue Fu, Han Liu, Shuting Liang, Mingming Song, Aining Zhang, Dong‐en Wang, Qianjin Guo, You Zhang, Jiawei Wu, Huiyun Xu

**Affiliations:** ^1^ School of Medicine Northwest University Xi'an Shaanxi China; ^2^ Key Laboratory for Space Bioscience and Biotechnology, School of Life Science and Technology Northwestern Polytechnical University Xi'an Shaanxi China; ^3^ College of Life Sciences Northwest University Xi'an Shaanxi China; ^4^ Division of Endocrinology, Department of Medicine Stanford University Stanford California USA

**Keywords:** aged tibiae, anabolism, Cx43 hemichannel, mechanical loading, Piezo1

## Abstract

Bone mechanosensitivity declines with age, resulting in a reduced anabolic response to mechanical stimulation. Although the mechanosensitive ion channel Piezo1 plays a critical role in bone mechanoresponsiveness, its mechanisms of mechanotransduction in aged bone remain incompletely understood. Here, we report that aged tibiae from 19‐month‐old male mice exhibit a blunted anabolic response to axial cyclic compressive loading or administration of the Piezo1 agonist Yoda1 alone. However, chemical activation of osteocytic Piezo1 by Yoda1 effectively rescues the anabolic response of aged cortical bone to mechanical loading. Mechanistically, Yoda1‐induced Piezo1 activation promotes its co‐localization with connexin 43 (Cx43) on the osteocyte membrane in response to mechanical stimulation. This Piezo1‐Cx43 interaction enhances Cx43 hemichannel (HC) activity via the PI3K‐Akt signaling pathway. Subsequent HC opening facilitates the release of the anabolic factor prostaglandin E_2_ (PGE_2_) and suppresses the catabolic factor sclerostin (SOST), thereby promoting bone formation and inhibiting bone resorption on the endosteal surface, which ultimately increases cortical bone mass during mechanical loading. Collectively, our findings reveal a mechanism whereby Piezo1 mediates the skeletal anabolic response to mechanical loading in aged bone via Cx43 HCs, offering new perspectives for combating age‐related osteoporosis.

## Graphical abstract

Yoda1‐induced Piezo1 activation enhances Piezo1‐Cx43 interaction upon mechanical stimulation, triggering PI3K‐Akt signaling to drive Cx43 phosphorylation and hemichannel opening. These events elevate PGE_2_ release, suppress SOST expression in senescent osteocytes, and ultimately enhance bone anabolism on the endosteal surface.

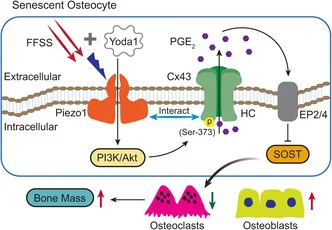

## 1 Introduction

Bone is a highly dynamic tissue that undergoes continuous remodeling to adapt to mechanical environments. Mechanical loading serves as a potent anabolic stimulus that enhances bone mass and maintains skeletal strength, whereas insufficient mechanical stimulation leads to bone loss (Rolvien and Amling 2022). Skeletal aging is associated with a marked decline in mechanosensitivity, which is closely linked to age‐related osteoporosis. Evidence indicates that aging blunts the osteogenic response to loading, attenuating the upregulation of bone‐forming genes and limiting bone mass accrual (Chermside‐Scabbo et al. 2020; Meakin et al. 2014). In addition, aging diminishes the capacity of mechanical stimulation to rescue disuse‐induced bone loss (Cunningham et al. 2023). Therefore, elucidating the mechanisms underlying diminished mechanoresponsiveness in aging is crucial for developing novel therapeutic strategies for age‐related osteoporosis.

Osteocytes, the most abundant cell type in bone tissue, are key mechanosensors that regulate skeletal adaptation to mechanical stimulation. However, their mechanoresponsiveness is markedly attenuated with senescence; for instance, intracellular Ca^2+^ oscillations, an early response of osteocytes to mechanical stimulation, are delayed and exhibit reduced amplitude in aged tibiae compared to young controls (Morrell et al. 2020). Moreover, mechanical loading fails to robustly suppress the expression of sclerostin (SOST), a Wnt signaling inhibitor, in osteocytes from aged mice (Holguin et al. 2016). Primary osteocytes isolated from aged mice show significantly reduced plasma membrane mechanosensitivity (Hagan et al. 2020) and decreased secretion of prostaglandin E_2_ (PGE_2_), an important anabolic mediator (Chalil et al. 2015). Therefore, strategies to enhance osteocyte mechanosensitivity could restore bone anabolic responses in aging.

Piezo1, a key mechanosensitive ion channel in osteocytes, is essential for transducing mechanical signals in bone (Yang et al. 2022). In vitro studies demonstrate that Piezo1 activation triggered by fluid shear stress (FSS) (Li et al. 2019; Lin et al. 2022) or vibrational stimulation upregulates Wnt1 expression through the YAP/TAZ pathway (Lin et al. 2025) and enhances production of anti‐osteoclastogenic protein osteoprotegerin (OPG) in osteocytes via the calmodulin/mTOR pathway (Li et al. 2023; Liu et al. 2022). Additionally, mechanical stretch suppresses the expression of catabolic factor SOST in osteocytes by activating the Piezo1‐Akt pathway (Sasaki et al. 2020). Notably, Piezo1 overexpression in osteocytes further augments mechanically induced synthesis of PGE_2_ (Li et al. 2019). All the mechano‐activated responses can be pharmacologically mimicked in osteocytes using Yoda1, a small‐molecule Piezo1 agonist (Li et al. 2019, 2023; Lin et al. 2022, 2025; Liu et al. 2022; Sasaki et al. 2020). In vivo, osteocyte‐specific Piezo1 deletion in adult mice impairs loading‐induced bone formation in tibiae and ulnae (Hendrickx et al. 2021; Li et al. 2019). Conversely, pharmacological activation of Piezo1 in osteocytes via Yoda1 promotes bone mass accrual in adult mice (Li et al. 2019). Of note, Piezo1 expression declines with age in both murine and human cortical bone (Li et al. 2023; Sun et al. 2019), and its loss in osteocytes exacerbates age‐related cortical bone loss (Li et al. 2023), suggesting that Piezo1 is involved in the senescence‐associated decline in osteocyte mechanosensitivity. Recent studies have shown that combining mechanical loading with Yoda1 (or its analog Yoda2) additively improves cortical bone parameters in 50‐week‐old (Wasi et al. 2024) and 22‐month‐old female mice (Meslier et al. 2025). Moreover, in a model of breast cancer‐induced osteolysis, combined tibial loading and Yoda1 treatment conferred superior skeletal protection in aged mice compared to monotherapies (Wasi et al. 2025). However, specific mechanisms by which Piezo1 regulates mechanical responses in aged bone remain incompletely understood.

Connexin 43 (Cx43) is the most abundantly expressed connexin subtype in osteocytes and forms mechanosensitive hemichannels (HCs). Mechanical stimulation promotes a conformational change of integrin α5 on osteocytes (Batra, Riquelme, Burra, Kar, et al. 2014; Riquelme et al. 2021), which subsequently triggers the opening of Cx43 HCs (Batra et al. 2012). Once opened, Cx43 HCs facilitate the release of small‐molecule bone anabolic factors (≤ 1.2 kDa) from osteocytes into the extracellular microenvironment (Zhao et al. 2024). Our previous studies have shown that mechanically induced Cx43 HC opening stimulates the release of PGE_2_ from osteocytes, which acts in an autocrine/paracrine manner to suppress the expression of SOST in osteocytes and promote endosteal bone formation (Zhao, Hua, et al. 2022; Zhao, Riquelme, et al. 2022). In contrast, inhibition of Cx43 HCs impedes the anabolic response to mechanical loading (Zhao, Riquelme, et al. 2022). However, in aged mice, osteocytes exhibit reduced Cx43 expression (Tu et al. 2022). Notably, we recently demonstrated that targeted activation of HCs using a Cx43‐specific antibody restores the impaired anabolic response to mechanical loading in 22‐month‐old male mice (Zhao et al. 2024).

The opening of Cx43 HCs in osteocytes is closely associated with Piezo1 activation (Zeng et al. 2022). Upon FSS stimulation, activated Piezo1 enhances its membrane colocalization with Cx43 and promotes Cx43 HC opening via the PI3K‐Akt signaling pathway (Iyyathurai et al. 2018; Zeng et al. 2022). The opening of Cx43 HCs facilitates ATP release, which in turn activates purinergic receptors, amplifies Ca^2+^ influx, and further sustains Cx43 HC activity (Zeng et al. 2022). Conversely, Piezo1 inhibition completely impedes the opening of Cx43 HCs (Zeng et al. 2022). Moreover, Cx43 knockout is associated with reduced Piezo1 protein expression (Zeng et al. 2022), suggesting a reciprocal interaction relationship. However, whether this interaction between Piezo1 and Cx43 participates in mechanically insensitive aged bone remains unknown.

In this study, we investigate the specific role of the Piezo1‐Cx43 HC axis in regulating anabolic responses within mechanically insensitive aged bone. We hypothesized that Piezo1 serves as an upstream regulator of Cx43 HCs in osteocytes. Specifically, chemical activation of Piezo1 under mechanical stimulation may enhance its interaction with Cx43 to promote the opening of the Cx43 HCs, thereby rescuing the anabolic responses of aged bone. Our results showed that the combined treatment significantly enhanced Piezo1‐Cx43 interaction, promoted Cx43 HC opening via the PI3K‐Akt signaling, and led to increased PGE_2_ release coupled with suppressed SOST expression in osteocytes. Consequently, endosteal bone anabolism was robustly potentiated. Given the current lack of effective anabolic therapies for aged and disuse‐related osteoporosis, co‐activation of Piezo1 with moderate mechanical loading presents a promising translational strategy for patients with diminished mechanosensitivity.

## 2 Results

### 2.1 Aging Blunts the Anabolic Responses of Tibial Bone to Mechanical Loading

**FIGURE 1:**
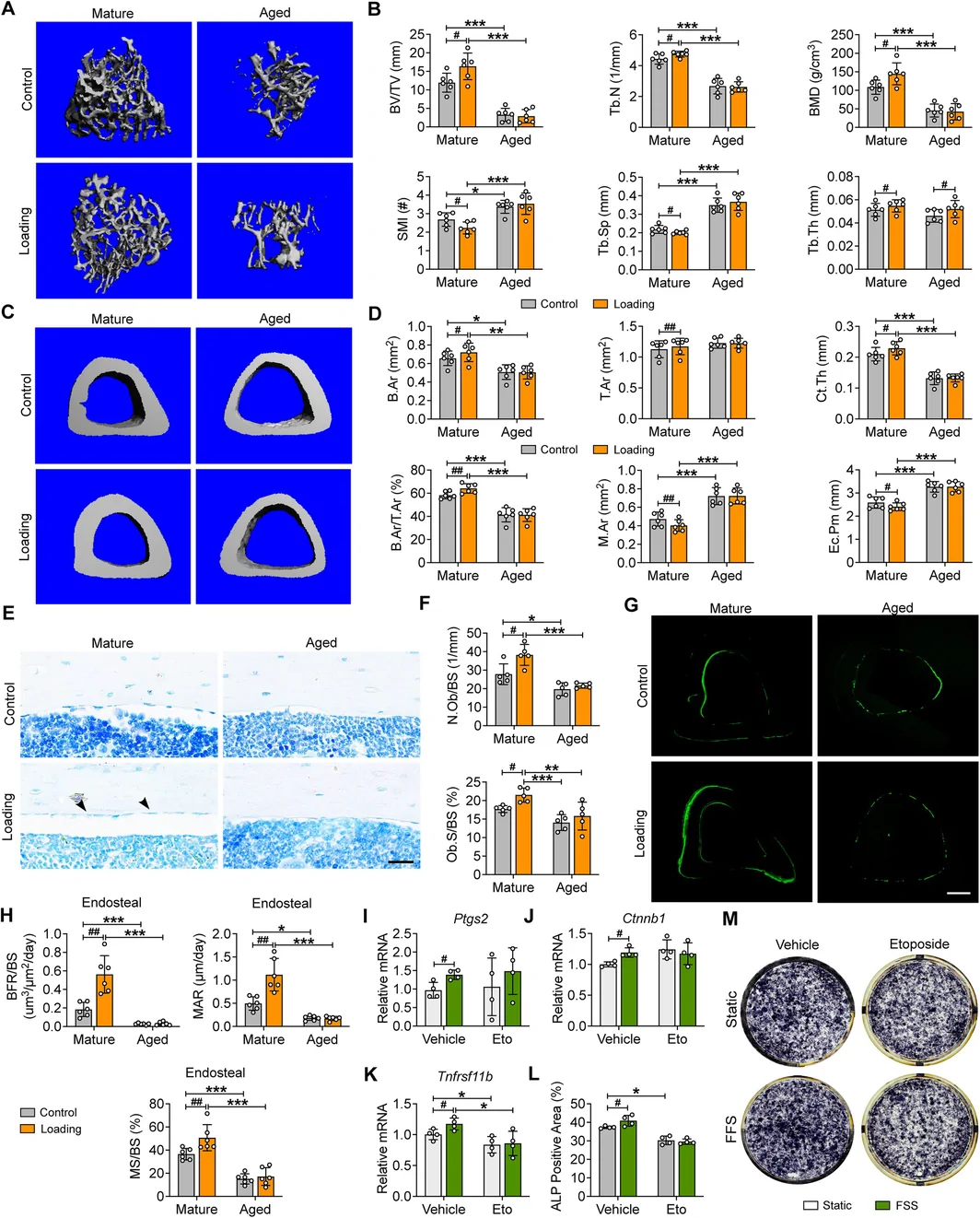
Aging blunts the anabolic responses of tibial bone to mechanical loading. (A–D) Representative μCT 3D images showing the metaphyseal trabecular bone (A) and mid‐diaphyseal cortical bone (C) in mature and aged mice, and the corresponding quantitative analysis results (B and D). *n* = 6. (E) Toluidine blue staining labels osteoblasts (black arrows) on the endosteal surfaces of the mid‐diaphysis. Scale bar, 25 μm. (F) Quantitative analysis of the osteoblast number per millimeter of bone surface (N.Ob/BS) and osteoblast surface per bone surface (Ob.S/BS). *n* = 5. (G) Calcein double labeling of the mid‐diaphysis in mature and aged mice. Scale bar, 100 μm. (H) Dynamic histomorphometric analysis of bone formation on the endosteal surface. *n* = 6. (I–K) qRT‐PCR analysis of gene expression in etoposide‐treated MLO‐Y4 cells under FSS stimulation. *n* = 4. (L, M) ALP staining of MC3T3‐E1 cells cultured in conditioned medium from normal or senescent MLO‐Y4 cells in response to FSS stimulation, and the corresponding quantification. *n* = 4. Data are expressed as mean ± SD. (B, D, F, H) ^#^ *p* < 0.05 and ^##^ *p* < 0.01 by the paired Student's *t*‐test. (I–L) ^#^ *p* < 0.05 by unpaired Student's *t*‐test. (B, D, F, H–L) *p < 0.05, **p < 0.01, and ***p < 0.001 by two‐way ANOVA with Tukey test. Eto, etoposide.

To assess the impact of aging on bone's adaptive response to mechanical loading, we applied a 2‐week regimen of cyclic axial tibial compression (1200 με) to the left tibiae of 16‐week‐old mature male mice and 19‐month‐old aged male mice, with the right tibiae serving as non‐loaded contralateral controls (Figure S1A–C). μCT revealed a significant age‐related decline in bone mass at both the metaphyseal trabecular bone and cortical mid‐diaphysis (50% distal from the proximal end) in non‐loaded contralateral controls from aged mice compared to mature mice (Figure 1A–D). In mature mice, mechanical loading markedly improved trabecular architecture, reflected by increased bone volume fraction (BV/TV), trabecular number (Tb.N), bone mineral density (BMD), and structural model index (SMI), alongside decreased trabecular separation (Tb.Sp) compared to contralateral controls (Figure 1A,B). In contrast, aged mice exhibited a complete absence of these anabolic responses in the trabecular compartment under mechanical loading. Notably, trabecular thickness (Tb.Th) was increased by loading in both mature and aged mice (Figure 1B). A similar attenuation of anabolic response to tibial loading was observed in aged cortical bone at the mid‐diaphysis. The cortical bone parameters, including bone area (B.Ar), cross‐sectional area (T.Ar), bone area ratio (B.Ar/T.Ar), cortical thickness (Ct.Th), bone marrow area (M.Ar), and endocortical perimeter (Ec.Pm) remained unaltered by 2‐week tibial loading in aged mice (Figure 1C,D). In contrast, compared to contralateral controls, mechanical loading significantly increased B.Ar, T.Ar, and Ct.Th in mature mice (Figure 1D). Furthermore, the elevated B.Ar/T.Ar was driven by a reduction in marrow cavity size, as evidenced by decreased M.Ar and Ec.Pm (Figure 1D).

The reduced M.Ar in loaded tibiae suggested altered remodeling on the endosteal surface. Consistently, cortical histomorphometry at the mid‐diaphysis (50% site) revealed that mechanical loading significantly increased the number of osteoblasts on endosteal surface between the loaded and contralateral tibiae in mature mice (Figure 1E,F), leading to elevated bone formation rate (BFR/BS), mineral apposition rate (MAR), and mineralizing surface per surface BS (MS/BS) (Figure 1G,H). In contrast, aged mice exhibited no significant differences with respect to the number of osteoblasts and bone formation parameters on the endosteal surface between the loaded and contralateral tibiae (Figure 1E–H). Given that impaired anabolic responses in aged bone are associated with osteocyte senescence (Qin et al. 2020), we asked whether inducing senescence directly reduces mechanosensitivity in osteocytes. The osteocyte‐like cell line MLO‐Y4 was treated with 5 μM etoposide for 24 h, a topoisomerase inhibitor that induces DNA damage and cellular senescence (Moiseeva et al. 2023; Petrova et al. 2016). Etoposide‐treated MLO‐Y4 cells exhibited established senescence markers, including increased β‐galactosidase activity (Figure S2A–D) and elevated expression of senescent genes *p16*, *p21*, and *p53* (Figure S2E). We further found that etoposide treatment abolished FSS‐induced upregulation of COX‐2 (*Ptgs2*), β‐catenin (*Ctnnb1*), and *OPG* (*Tnfrsf11b*) expression, three well‐known mechanoresponsive genes in osteocytes (Zhao et al. 2024) (Figure 1I–K). Moreover, the conditioned medium collected from FSS‐stimulated MLO‐Y4 cells promoted the alkaline phosphatase (ALP) level in the osteoblast‐like cell line MC3T3‐E1. Contrastingly, the conditioned medium from senescent MLO‐Y4 cells subjected to fluid flow failed to promote osteogenic differentiation (Figure 1L,M). Collectively, these results indicate that aging diminished the anabolic response of tibiae to mechanical loading.

### 2.2 Chemical Activation of Piezo1 Using Yoda1 Promotes Load‐Induced Cortical Anabolism in Aged Tibiae

**FIGURE 2:**
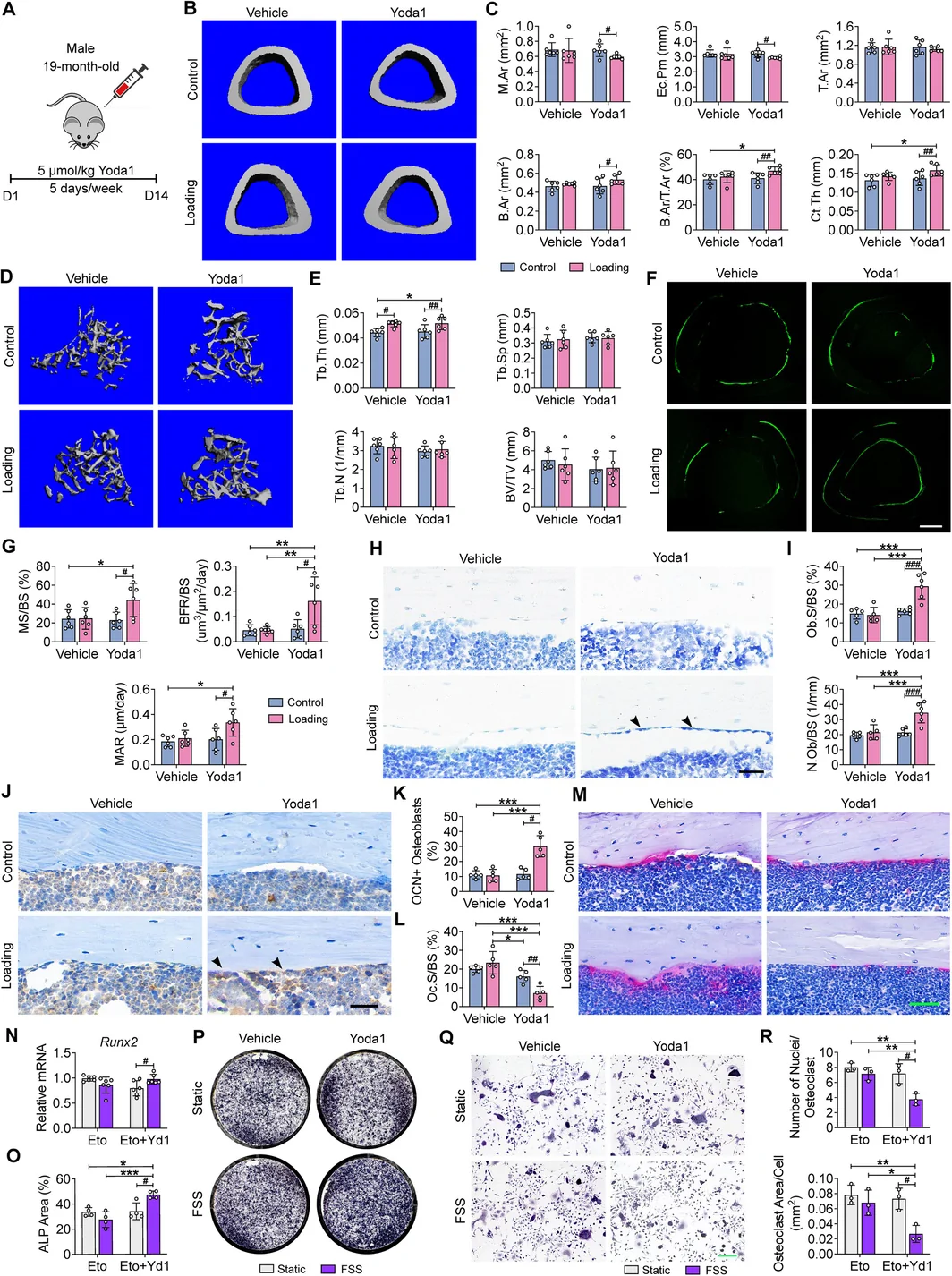
Chemical activation of Piezo1 with Yoda1 promotes load‐induced cortical anabolism in aged tibiae. (A) Schematic diagram of Yoda1 treatment. (B–E) Representative 3D images and quantitative analysis of mid‐diaphyseal cortical bone and metaphyseal trabecular bone in vehicle‐ and Yoda1‐treated aged mice. *n* = 6. (F, G) Representative calcein double labeling of the mid‐diaphysis and corresponding quantitative analysis on the endosteal surface in vehicle‐ and Yoda1‐treated aged mice. Scale bar, 100 μm. *n* = 6. (H, I) Toluidine blue staining of osteoblasts (blue; black arrows) on endosteal surfaces at the mid‐diaphysis. Scale bar, 25 μm. *n* = 6. (J, K) Immunohistochemical staining for osteocalcin (OCN) labeling osteoblasts (brown; black arrows) on endosteal surfaces in vehicle‐ and Yoda1‐treated aged mice, and corresponding quantitative analysis. Scale bar, 40 μm. *n* = 6. (L, M) TRAP staining of osteoclasts on the endosteal surface, and the corresponding quantification of osteoclast surface per millimeter of bone surface (Oc.S/BS). Scale bar, 30 μm. *n* = 6. (N–P) ALP staining and qRT‐PCR analysis evaluating the effects of conditioned medium from senescent MLO‐Y4 cells treated with Yoda1 under FSS stimulation on the osteogenic differentiation of MC3T3‐E1 cells. *n* = 4–5. (Q, R) TRAP staining showing the effects of conditioned medium from senescent MLO‐Y4 cells on the differentiation of RAW264.7 cells. Scale bar, 200 μm. *n* = 3. Data are expressed as mean ± SD. (C, E, G, I, K, L) ^#^ *p* < 0.05, ^##^ *p* < 0.01, and ^###^ *p* < 0.001 by the paired Student's *t*‐test. (N, Q, R) ^#^ *p* < 0.05 by unpaired Student's *t*‐test. (C, E, G, I, K, L, N, O, R) *p < 0.05, **p < 0.01, and ***p < 0.001 by two‐way ANOVA with Tukey test. Eto, etoposide; Yd1, Yoda1.

Subsequently, we investigated whether pharmacological activation of Piezo1 could rescue the blunted anabolic response to mechanical loading in aged tibiae. To this end, 19‐month‐old aged male mice were administered 5 μmol/kg Yoda1, a known agonist that lowers the mechanical threshold for Piezo1 activation (Botello‐Smith et al. 2019) (Figure 2A). Yoda1 administration did not alter body weight (Figure S3A). Histopathological assessment of major visceral organs confirmed that all mice remained in good health throughout the loading experiment at this dosage (Figure S3B,C). μCT analysis revealed that Yoda1 treatment alone exerted no significant effects on mid‐diaphyseal cortical bone architecture in aged male mice, with no significant differences observed between vehicle‐control and Yoda1‐control tibiae (Figure 2B,C). Notably, in Yoda1‐treated mice, the loaded tibiae exhibited a reduction in bone marrow cavity size (as indicated by M.Ar and Ec.Pm) relative to the contralateral tibiae, accompanied by significant increases in B.Ar, B.Ar/T.Ar, and Ct.Th (Figure 2C). In contrast, Yoda1 treatment did not influence the trabecular bone responsiveness to tibial loading, as evidenced by unaltered Tb.Th, Tb.Sp, Tb.N, and BV/TV in the loaded tibiae of Yoda1‐treated aged mice (Figure 2D,E). Thus, we focus on cortical anabolic responsiveness to mechanical loading. Dynamic histomorphometry demonstrated significant enhancements in MS/BS, BFR/BS, and MAR on the endosteal surface of loaded tibiae from Yoda1‐treated aged mice, which were not observed in vehicle‐treated aged mice (Figure 2F,G). Consistent with these findings, toluidine blue staining and immunohistochemistry revealed a marked increase in osteoblast number and osteocalcin (OCN) protein expression on the endosteal surface following mechanical loading in Yoda1‐treated aged mice (Figure 2H–K). Furthermore, TRAP staining revealed a significant reduction in osteoclast activity on the endosteal surface of loaded tibiae from Yoda1‐treated mice (Figure 2L,M). We next sought to determine whether osteocytes contribute to the Yoda1‐induced enhancement of bone mechanoresponsiveness in aged mice. Following treatment with etoposide, Yoda1‐exposed MLO‐Y4 cells were subjected to FSS stimulation, and thereafter the conditioned medium was applied to MC3T3‐E1 osteoblastic cells and RAW264.7 osteoclastic cells (Figure S4A). The conditioned medium collected from Yoda1 + FSS senescent MLO‐Y4 cells promoted a significant enhancement of osteoblast differentiation, as indicated by the upregulated gene *Runx2* expression (Figure 2N), along with increases in ALP level (Figure 2O,P). In addition, the same conditioned medium markedly suppressed osteoclast differentiation (Figure 2Q,R). In line with these observations, Yoda1 treatment significantly reduced the gene expression of RANKL (*Tnfsf11*) in response to FSS stimulation in senescent MLO‐Y4 cells (Figure S5A). Taken together, these findings suggest that Piezo1 activation via Yoda1 improves mechanical adaptation in aged male bone by modulating osteocyte‐mediated coupling between osteoblast and osteoclast activity.

To assess whether Piezo1 activation exerts a similar effect on the anabolic response to mechanical loading in aged females, 19‐month‐old female mice treated with Yoda1 were subjected to 2 weeks of tibial loading at 1200 με (Figures S3D and S6A). μCT analysis showed very little trabecular bone in aged female mice, causing significant increases in BV/TV and Tb.N in the vehicle group (Figure S6B,C). In the mid‐diaphyseal region, tibial loading alone failed to induce significant alterations in cortical bone parameters (Figure S6D,E). Yoda1 treatment alone showed a trend toward higher Ct.Th and B.Ar/T.Ar and lower M.Ar relative to vehicle controls (Figure S6D,E). Consistent with the response seen in aged male mice, the combination of Yoda1 administration and mechanical loading significantly enhanced these cortical bone parameters (Figure S6D,E), which was attributed to increased endosteal bone formation (Figure S6F,G). Thus, the effect of Piezo1 activation on the anabolic response to mechanical loading exhibited no obvious sex difference.

### 2.3 Yoda1 Treatment Enhances the Interaction of Piezo1 and Cx43, Promoting the Opening of Cx43 HCs Under Mechanical Stimulation in Senescent Osteocytes

**FIGURE 3:**
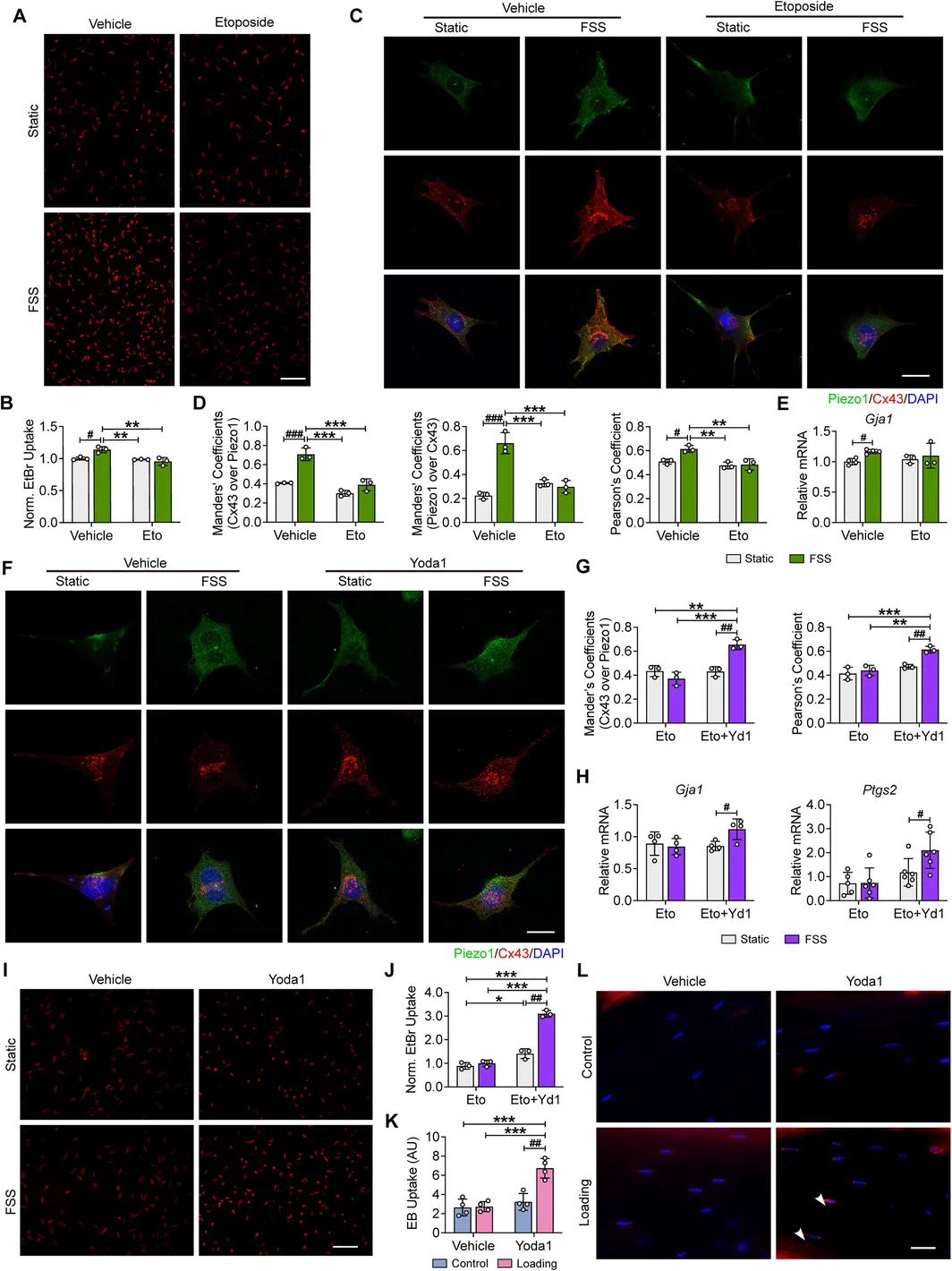
Yoda1 treatment enhances the colocalization of Piezo1 and Cx43, promoting the opening of Cx43 HCs under mechanical stimulation in senescent osteocytes. (A, B) Representative images and corresponding quantification of EtBr dye uptake levels in normal and senescent MLO‐Y4 cells in response to FSS stimulation. Scale bar, 200 μm. *n* = 3. (C, D) Immunofluorescence images showing Piezo1 and Cx43 colocalization on the normal and senescent MLO‐Y4 cell surface under FSS stimulation, and the corresponding quantification of colocalization levels. Scale bar, 20 μm. *n* = 3. (E) *Gja1* gene expression in normal and senescent MLO‐Y4 cells under FSS stimulation. *n* = 3–4. (F, G) Immunofluorescence of Piezo1 and Cx43 colocalization on the senescent MLO‐Y4 cell surface treated with Yoda1 in response to FSS stimulation, and the corresponding colocalization quantification levels. Scale bar, 20 μm. *n* = 3. (H) *Gja1* and *Ptgs2* gene expression in senescent MLO‐Y4 cells treated with Yoda1 under FSS stimulation. *n* = 4–6. (I, J) The EtBr dye uptake level in senescent MLO‐Y4 cells treated with Yoda1. Scale bar, 200 μm. *n* = 3. (K, L) Representative fluorescence images and quantitative analysis of in situ EB uptake by osteocytes (white arrowheads) in mid‐diaphysis of loaded and contralateral tibiae from vehicle‐ and Yoda1‐treated aged mice. Scale bar: 20 μm. *n* = 4. Data are expressed as mean ± SD. (B, D, E, G, H, J) ^#^ *p* < 0.05, ^##^ *p* < 0.01, and ^###^ *p* < 0.001 by unpaired Student's *t*‐test. (K) ^##^ *p* < 0.01 by the paired Student's *t*‐test. (B, D, E, G, H, J, K) *p < 0.05, **p < 0.01, and ***p < 0.001 by two‐way ANOVA with Tukey test. Eto, etoposide; Yd1, Yoda1.

Based on the established role of Piezo1 in regulating the opening of HCs in mature bone (Zeng et al. 2022), we proceeded to investigate whether Yoda1‐induced mechanical adaptation in aged bone involves Cx43 HC activity. HC opening was assessed in vitro by measuring ethidium bromide (EtBr) uptake in osteocytes (Zeng et al. 2022; Zhao, Riquelme, et al. 2022). We detected that FSS stimulation significantly increased EtBr fluorescence in normal MLO‐Y4 cells, whereas etoposide‐induced senescence blunted this HC response to FSS stimulation (Figure 3A,B). Notably, no difference in EtBr fluorescence was observed between unstimulated normal and senescent osteocytes, suggesting that the opening of Cx43 HCs is dependent on mechanical stimulation (Figure 3B). Confocal immunofluorescence revealed that FSS stimulation enhanced the colocalization of Piezo1 and Cx43 on the plasma membrane of normal osteocytes, as reflected by markedly increased Mander's and Pearson's coefficients (Figure 3C,D). By contrast, this enhanced colocalization was absent in senescent MLO‐Y4 cells (Figure 3C,D). Correspondingly, the FSS‐induced upregulation of Cx43 (*Gja1*) expression was also blunted in senescent cells (Figure 3E). We then treated senescent osteocytes with Yoda1. While Yoda1 alone had no observable effects on Piezo1‐Cx43 colocalization between unstimulated senescent and normal MLO‐Y4 cells, it significantly increased colocalization in senescent cells under FSS stimulation (Figure 3F,G). In situ proximity ligation assay (PLA) further confirmed the enhanced interaction between Piezo1 and Cx43 on the cell bodies of senescent MLO‐Y4 cells following Yoda1 + FSS treatment (Figure S7A,B). Furthermore, FSS stimulation significantly upregulated the expression of *Gja1* and *Ptgs2* in Yoda1‐treated senescent MLO‐Y4 cells, but not in vehicle‐treated senescent MLO‐Y4 cells (Figure 3H). Consistent with these findings, Yoda1 treatment restored FSS‐induced EtBr uptake in senescent osteocytes (Figure 3I,J). These in vitro EtBr uptake results were complemented by in situ assays, in which we examined Evans blue (EB) dye uptake by osteocytes in the mid‐diaphyseal cortical bone region of aged tibiae in response to mechanical loading, as previously described (Acosta et al. 2024; Zhao, Hua, et al. 2022; Zhao, Riquelme, et al. 2022; Zhao et al. 2024). We found that the EB fluorescence intensity was significantly increased in loaded tibiae of Yoda1‐treated aged mice, but not in those of vehicle‐treated aged mice (Figure 3K,L). In line with these findings, in situ PLA detected enhanced interaction between Cx43 and integrin α5 on the cell bodies of senescent MLO‐Y4 cells following combined treatment with Yoda1 and FSS (Figure S7C,D), which represents a critical step required for Cx43 HC opening (Batra et al. 2012; Batra, Riquelme, Burra, Kar, et al. 2014). Together, these findings suggest that Piezo1 activation by Yoda1 facilitates its interaction with Cx43 and promotes the opening of Cx43 HCs in response to mechanical stimulation.

### 2.4 Yoda1 Enhances Aged Bone Mechanoresponsiveness by Triggering PGE_2_ Release and SOST Suppression in Osteocytes

**FIGURE 4:**
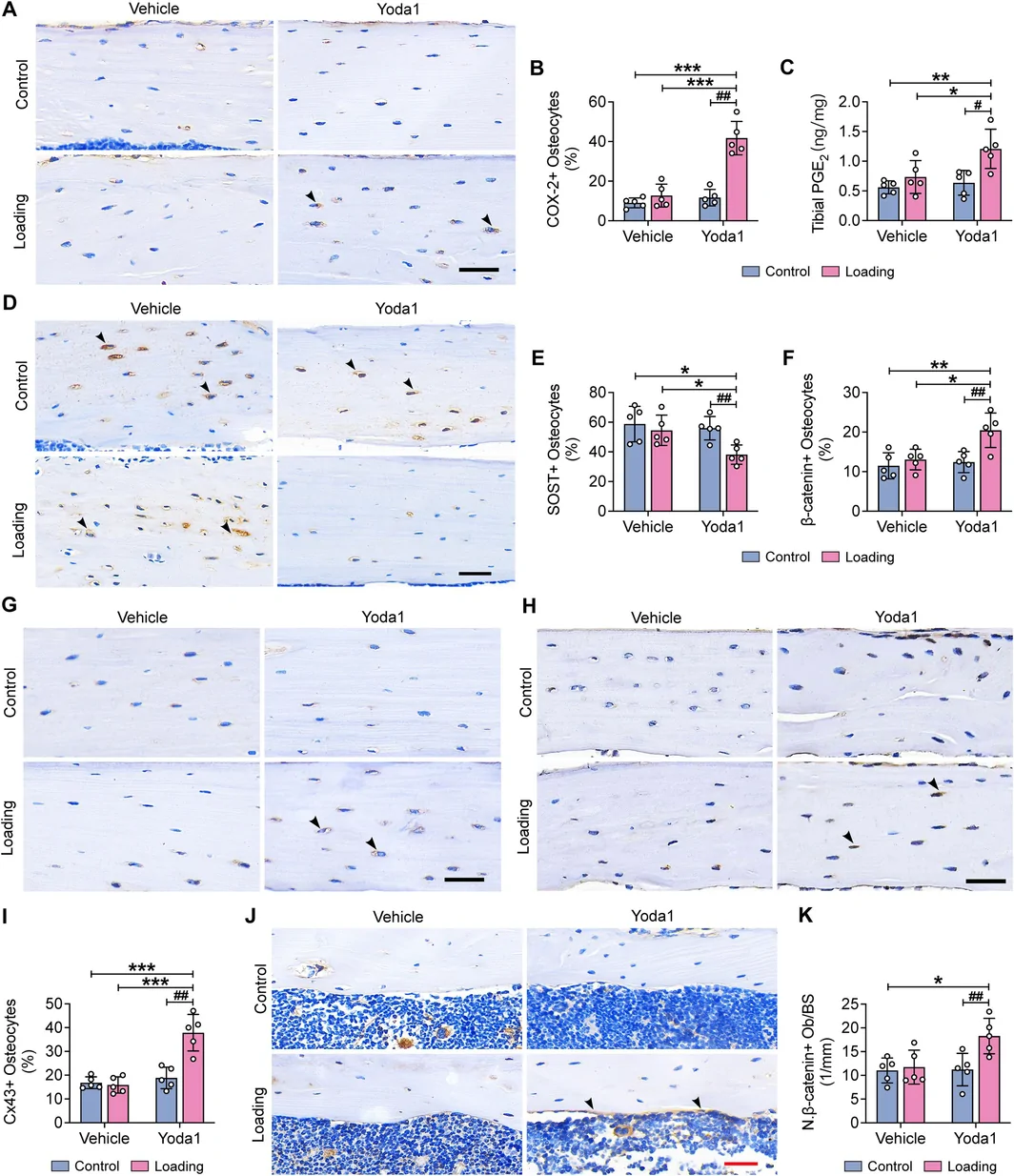
Yoda1 enhances aged bone mechanoresponsiveness by triggering PGE_2_ release and SOST suppression in osteocytes. (A, B) Representative COX‐2 immunohistostaining and quantification of COX‐2‐positive osteocytes in mid‐diaphyseal cortical bone from vehicle‐treated and Yoda1‐treated aged mice. Scale bar: 40 μm. *n* = 5. (C) PGE_2_ levels in bone marrow‐flushed tibial diaphysis in vehicle‐ and Yoda1‐treated aged mice. *n* = 5. (D–I) Immunohistochemical staining and corresponding quantification of SOST (D and E), β‐catenin (F and G), and Cx43 (H and I) in osteocytes (brown; black arrows) from mid‐diaphyseal cortical bone of vehicle‐ and Yoda1‐treated aged mice. Scale bar, 40 μm. *n* = 5. (J, K) Immunohistochemical staining and corresponding quantification of β‐catenin in osteoblasts (brown; black arrows) on endosteal surfaces in vehicle‐ and Yoda1‐treated aged mice. Scale bar, 40 μm. *n* = 5. Data are expressed as mean ± SD. (B, C, E, F, I, K) ^#^ *p* < 0.05 and ^##^ *p* < 0.01 by the paired Student's *t*‐test. (B, C, E, F, I, K) *p < 0.05, **p < 0.01, and ***p < 0.001 by two‐way ANOVA with Tukey test.

We subsequently examined metabolic changes in the bone cells within the mid‐diaphyseal cortical region of aged mice following mechanical loading. Consistent with the upregulated *Ptgs2* gene expression in Yoda1‐treated senescent MLO‐Y4 cells under FSS stimulation (Figure 3H), immunohistochemical staining revealed a significant increase in osteocytic COX‐2 protein expression in the loaded tibiae from Yoda1‐treated aged mice (Figure 4A,B). Moreover, 5‐day mechanical loading elevated PGE_2_ levels in the tibiae of Yoda1‐treated aged mice, an effect not observed in vehicle‐treated aged mice (Figure 4C). In Yoda1‐treated aged mice, there was a significant loading‐induced reduction in the expression of osteocytic SOST protein, a Wnt signaling antagonist (Figure 4D,E), while increasing stable, unphosphorylated β‐catenin expression in osteocytes (Figure 4F,G). Previous studies have shown that Cx43 expression and HC assembly are directly regulated by β‐catenin in osteocytes (Tu et al. 2012). Here, we observed a significant increase in Cx43‐positive osteocytes in loaded tibiae of Yoda1‐treated mice compared to contralateral non‐loaded controls (Figure 4H,I). Furthermore, Yoda1 treatment significantly increased unphosphorylated β‐catenin expression in endosteal osteoblasts following tibial loading (Figure 4J,K), an effect we have previously documented to be associated with anabolic responsiveness (Zhao, Hua, et al. 2022; Zhao, Riquelme, et al. 2022; Zhao et al. 2024). Contrastingly, none of these proteins, including that of COX‐2, SOST, Cx43, and β‐catenin, showed significant differences between loaded and contralateral tibiae in vehicle‐treated aged mice. These results indicate that the Yoda1‐induced improvement in the anabolic responsiveness to mechanical loading is driven by PGE_2_ release and subsequent SOST suppression in osteocytes, which promotes β‐catenin accumulation in both osteocytes and osteoblasts.

### 2.5 Piezo1‐Mediated Enhancement of Bone Mechanoresponsiveness Requires Cx43 HCs

**FIGURE 5:**
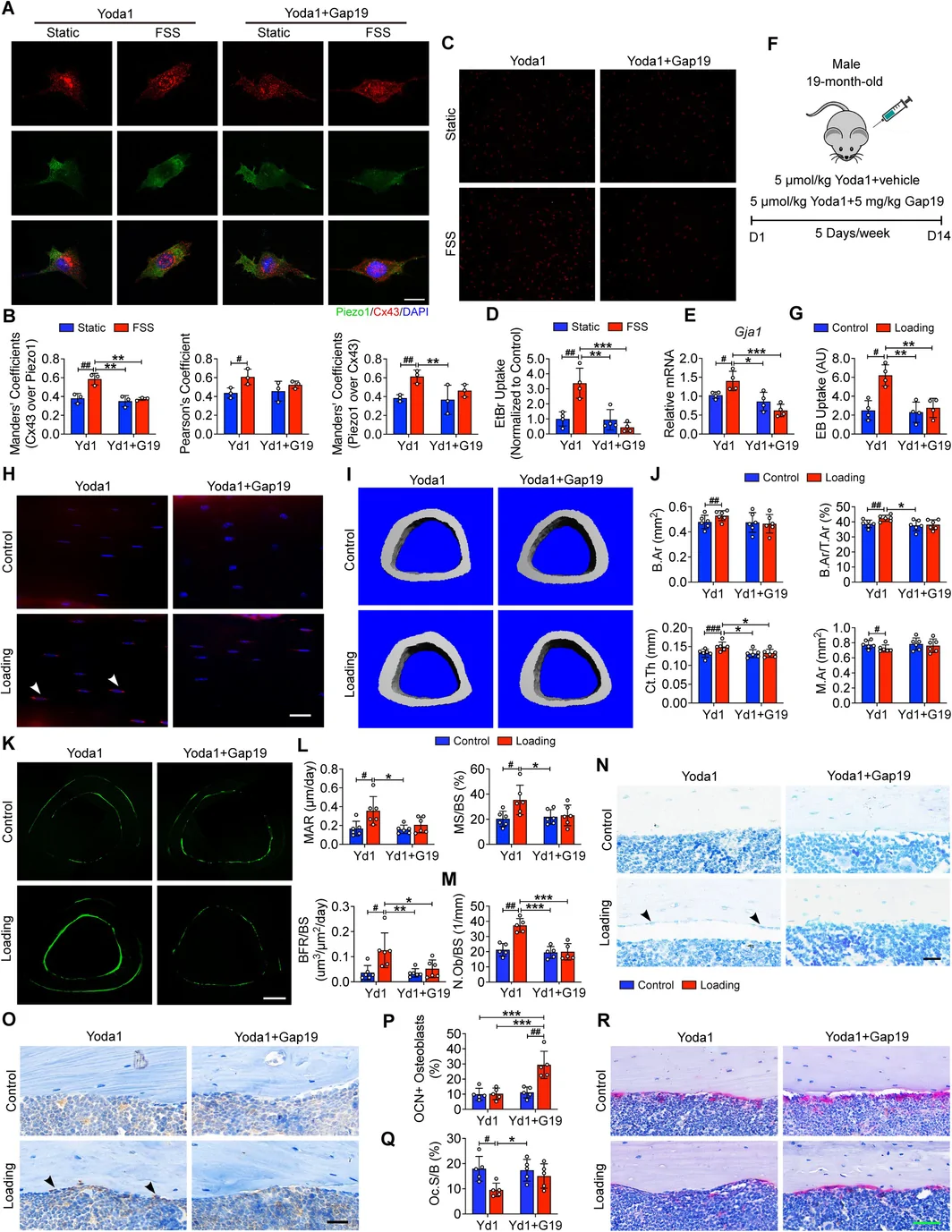
Piezo1‐mediated enhancement of bone mechanoresponsiveness requires Cx43 HCs. (A, B) Immunofluorescence images and corresponding quantification of Piezo1 and Cx43 colocalization on the senescent MLO‐Y4 cell surface treated with Yoda1 and Gap19 in response to FSS stimulation. Scale bar: 20 μm. *n* = 3. (C, D) Representative images of EtBr dye uptake in Yoda1‐ and Yoda1 + Gap19‐treated senescent MLO‐Y4 cells in response to FSS stimulation, and the corresponding quantification of EtBr uptake levels. Scale bar, 200 μm. *n* = 4. (E) *Gja1* gene expression in Yoda1‐ and Yoda1 + Gap19‐treated senescent MLO‐Y4 cells under FSS stimulation. *n* = 4. (F) Schematic diagram of Yoda1 and Gap19 treatments. (G, H) Representative fluorescence images of in situ EB dye uptake by osteocytes (white arrowheads) in the mid‐diaphysis of Yoda1‐ and Yoda1 + Gap19‐treated aged mice, and the corresponding quantification. Scale bar: 20 μm. *n* = 4. (I, J) μCT analysis of cortical bone architecture in mid‐diaphyseal tibiae. *n* = 6. (K, L) Calcein double labeling of the mid‐diaphysis. Scale bar, 100 μm. *n* = 6. (M, N) Toluidine blue staining of osteoblasts (brown; black arrows) on endosteal surfaces at the mid‐diaphysis. Scale bar, 25 μm. *n* = 5. (O, P) OCN immunohistochemical staining of osteoblasts (black arrows) on endosteal surfaces and the corresponding quantification. Scale bar, 40 μm. *n* = 6. (Q, R) TRAP staining of osteoclasts on endosteal surfaces and the corresponding quantification. Scale bar, 30 μm. *n* = 5. Data are expressed as mean ± SD. (B, D, E) ^#^ *p* < 0.05 and ^##^ *p* < 0.01 by unpaired Student's *t*‐test. (G, J, L, M, P, Q) ^#^ *p* < 0.05, ^##^ *p* < 0.01, and ^###^ *p* < 0.001 by the paired Student's *t*‐test. (B, D, E, G, J, L, M, P, Q) *p < 0.05, **p < 0.01, and ***p < 0.001 by two‐way ANOVA with Tukey test. Yd1, Yoda1; G19, Gap19.

To further investigate whether Piezo1‐mediated bone mechanoadaptation in aged mice involves Cx43 HC function, we utilized Gap19, a specific Cx43 HC inhibitory peptide that binds directly to the C‐terminal (CT) domain of Cx43 and blocks Cx43 HCs in osteocytes (Quan et al. 2022). In vitro, Gap19 treatment alone did not affect the basal colocalization of Piezo1 and Cx43 or HC activity in MLO‐Y4 cells (Figure 5A–D). However, Gap19 significantly suppressed both the FSS‐induced Piezo1‐Cx43 colocalization and HC opening in Yoda1‐treated senescent MLO‐Y4 cells, along with the associated upregulation of *Gja1* expression (Figure 5A–E). For in vivo evaluation, Gap19 was co‐administered intraperitoneally with Yoda1 in 19‐month‐old aged male mice for five consecutive days per week over the 2‐week tibial loading period (Figure 5F). The co‐administration had no significant effect on body weight and showed no drug‐related toxicity in visceral organs (Figure S3A–C). Consistent with the in vitro findings, in situ EB dye uptake assays revealed that the loading‐induced increase in osteocyte dye uptake observed in Yoda1‐treated aged male mice was abolished by Gap19 co‐treatment (Figure 5G,H). μCT analysis of mid‐diaphysis indicated that Gap19 alone did not alter cortical bone architecture (Figure 5I,J). However, whereas cyclic loading in Yoda1‐treated mice increased B.Ar, B.Ar/T.Ar, and Ct.Th while reducing M.Ar, these anabolic responses were absent in Yoda1 + Gap19‐treated mice (Figure 5I,J). Similarly, the loading‐induced increase in endosteal bone formation observed in Yoda1‐treated aged mice was suppressed by Gap19 (Figure 5K,L), as reflected by reduced osteoblast activity and lower OCN expression on the endosteal surface (Figure 5M–P). Furthermore, Gap19 also abrogated the loading‐induced reduction in osteoclast‐mediated bone resorption observed in Yoda1‐treated aged mice (Figure 5Q,R). Consistently, the inhibitory effect of Gap19 on anabolic mechanoresponsiveness was further confirmed in vitro. Gap19 suppressed the FSS‐induced upregulation of mechanoresponsive genes *Tnfrsf11b*, *Ptgs2*, and *Ctnnb1* seen in Yoda1‐treated senescent MLO‐Y4 cells (Figure S5B). Furthermore, compared to conditioned medium from Yoda1‐treated senescent MLO‐Y4 cells under FSS stimulation, medium collected from the Yoda1 + Gap19‐treated group showed no significant effect on osteoblast or osteoclast differentiation, as indicated by ALP‐positive area (Figure S8A,B) and TRAP‐positive cell counts (Figure S8C,D). Collectively, these findings demonstrate that osteocytic Cx43 HCs participate in the aged bone anabolic response to mechanical loading induced by Piezo1 activation. To further elucidate the role of Cx43 HCs in the mechanoresponsiveness of senescent osteocytes, we established an MLO‐Y4 cell line stably overexpressing *Gja1* through lentiviral transduction (Figure S9A,B). Etoposide‐induced senescent MLO‐Y4 cells overexpressing *Gja1* showed significantly enhanced HC opening under FSS stimulation over empty vector‐infected controls (Figure S9C,D), accompanied by increased gene expression of *ptgs2* and *Ctnnb1* (Figure S9E). Moreover, conditioned medium from FSS‐stimulated senescent MLO‐Y4 cells overexpressing *Gja1* promoted the alkaline phosphatase (ALP) level in the osteoblast‐like cell line MC3T3‐E1 osteoblastic cells, whereas medium from empty vector‐infected senescent MLO‐Y4 cells exhibited no such effect (Figure S9F,G).

### 2.6 Blocking Cx43 HCs Abolishes the Load‐Induced PGE_2_ Release and SOST Reduction in Osteocytes Observed in Yoda1‐Treated Aged Mice

We went on to examine metabolic changes in the bone cells within the mid‐diaphyseal cortical region following mechanical loading in Yoda1 + Gap19‐treated aged male mice. Compared with the Yoda1‐treated aged mice, mechanical loading no longer significantly altered tibial PGE_2_ levels in Yoda1 + Gap19‐treated mice (Figure S10A). Consistent with this, the loading‐induced increase in osteocytic COX‐2 protein expression observed in Yoda1‐treated aged mice was suppressed by Gap19 co‐administration (Figure S10B,C). Moreover, whereas tibial loading significantly reduced osteocytic SOST expression in Yoda1‐treated mice, no such reduction was observed in Yoda1 + Gap19‐treated mice (Figure S10D,E). In addition, Gap19 treatment abolished the loading‐induced increase in osteocytic unphosphorylated β‐catenin and Cx43 protein expression that was observed in Yoda1‐treated aged mice (Figure S10F–I). Furthermore, Gap19 administration significantly inhibited the osteoblastic expression of unphosphorylated β‐catenin observed in the loaded tibiae of Yoda1‐treated aged mice (Figure S10J,K). Notably, Gap19 did not significantly affect bone cell metabolism in contralateral non‐loaded tibiae. Interestingly, ploton silver staining revealed no significant difference in canalicular density between the loaded and control tibiae in either Yoda1‐treated or Yoda1 + Gap19‐treated mice (Figure S11A–D).

### 2.7 The PI3K‐Akt Signaling Pathway Mediates Piezo1‐Induced Mechanoresponsiveness in Aged Male Bone

**FIGURE 6:**
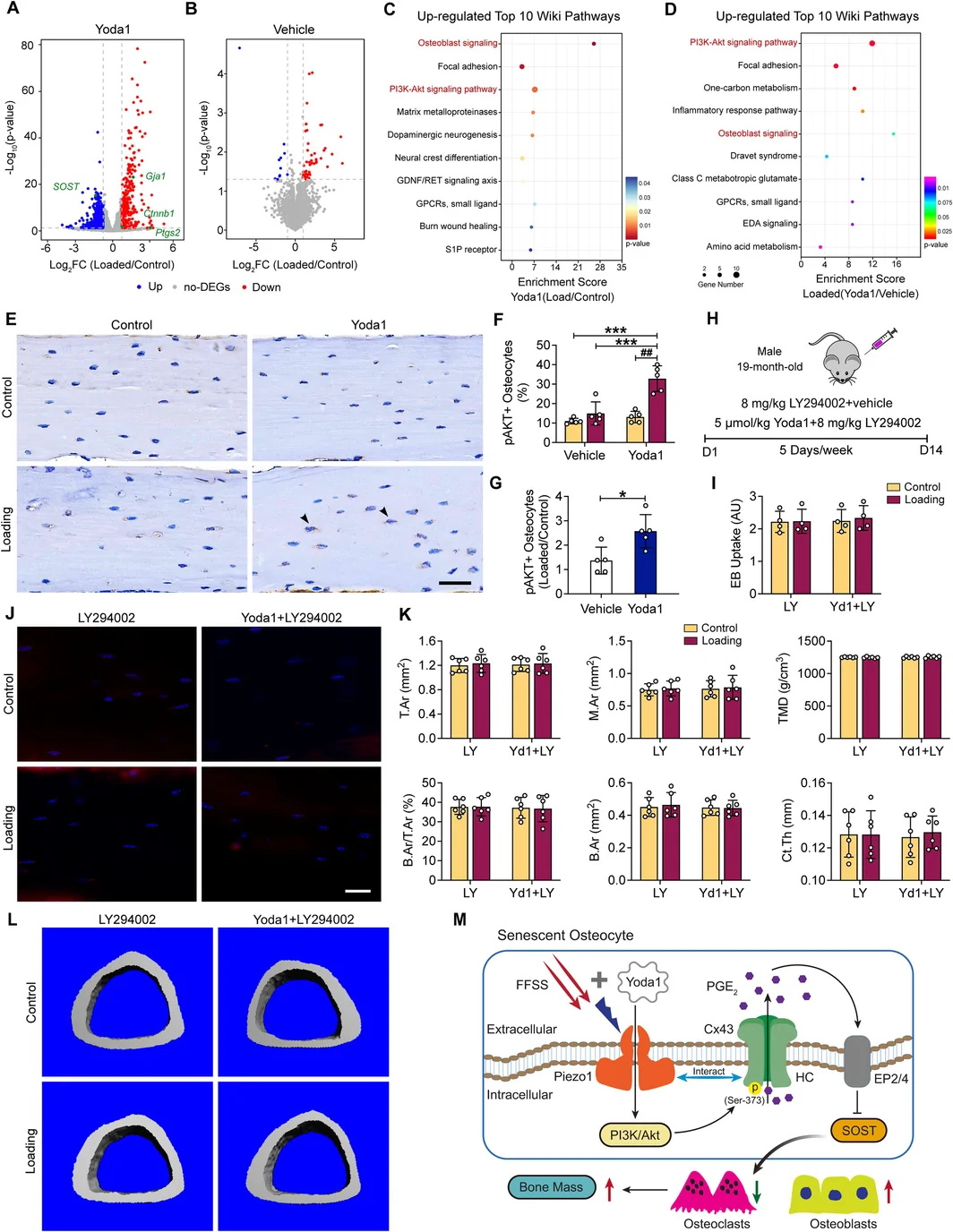
The PI3K‐Akt signaling pathway mediates Piezo1‐induced mechanoresponsiveness in aged bone. (A, B) Volcano plots showing the genes upregulated and downregulated in loaded versus contralateral tibiae from Yoda1‐ and vehicle‐treated aged mice. Blue and red dots represent significantly regulated genes (|log_2_ fold change| > 1, *p* value < 0.05). (C, D) Wikipathway analysis showing the top 10 enriched pathways between the loaded and contralateral tibiae in Yoda1 mice (C), or between the Yoda1 and vehicle treatment in loaded tibiae (D). (E) Representative immunohistostaining for phosphorylated Akt (pAkt) in mid‐diaphyseal cortical bone from vehicle‐ and Yoda1‐treated aged mice. Scale bar: 40 μm. (F, G) Quantification of pAkt‐positive osteocytes (F) and the ratio change (G). *n* = 5. (H) Schematic diagram of LY294002 treatments. (I, J) In situ EB dye uptake by osteocytes (white arrowheads) in the mid‐diaphysis of loaded and contralateral tibiae from Yoda1‐treated and Yoda1 + LY294002‐treated aged mice. Scale bar: 20 μm. *n* = 4. (K, L) Micro‐CT data of cortical bone architecture in mid‐diaphyseal tibiae. *n* = 6. (M) Schematic drawing of the mechanisms whereby activation of Piezo1 enhances the anabolic response to mechanical loading in aged tibiae via Cx43 HCs. Data are expressed as mean ± SD. (F) ^##^ *p* < 0.01 by the paired Student's *t*‐test. (G) *p < 0.05 by the unpaired Student's *t*‐test. (F) ***p < 0.001 by two‐way ANOVA with Tukey test. LY, LY294002; Yd1, Yoda1.

To further elucidate the mechanism through which Piezo1 activation by Yoda1 promotes load‐induced anabolism in aged male bone, we performed RNA sequencing (RNA‐seq) on mRNA isolated from tibial diaphyses stripped of periosteum and bone marrow. Transcriptomic analysis identified 652 differentially expressed transcripts in loaded versus nonloaded contralateral tibiae of Yoda1‐treated aged mice (Figure 6A). In contrast, vehicle‐treated aged mice failed to regulate the vast majority of mechanical loading‐sensitive transcripts (Figure S12A and Figure 6B). Gene ontology (GO) analysis and heatmap of DEGs indicated that the differentially expressed genes in Yoda1‐treated mice were enriched in terms associated with osteoblast differentiation and bone formation in loaded tibiae compared to non‐loaded controls, whereas most loading‐responsive transcripts remained unaltered in vehicle‐treated mice (Figure S12B,C). Gene‐set enrichment analysis (GSEA) showed significant enrichment of bone mineralization‐related signaling events in loaded tibiae of Yoda1‐treated aged mice relative to contralateral non‐loaded tibiae, suggesting enhanced osteoblast activity (Figure S12D). Kyoto Encyclopedia of Genes and Genomes (KEGG) pathway analysis further revealed upregulation of gap junction and Wnt signaling pathways in loaded tibiae of Yoda1‐treated aged mice, featuring the osteogenesis‐critical genes *Gja*1 and *Wnt1* (Figure S12E). Furthermore, Wikipathway enrichment analysis identified the PI3K‐Akt signaling pathway as the top‐enriched pathway both in loaded versus non‐loaded tibiae of Yoda1‐treated aged mice and in loaded tibiae between Yoda1‐treated and vehicle‐treated aged mice (Figure 6C,D, and Figure S12F). Consistent with RNA‐Seq findings, immunohistochemistry demonstrated a loading‐induced increase in phosphorylated Akt expression in osteocytes of Yoda1‐treated but not vehicle‐treated aged mice (Figure 6E–G). In silico analysis further defined Ser‐373, located at the C‐terminal domain of Cx43, as the predominant consensus Akt phosphorylation site on Cx43 (Figure S13A–E). Western blotting demonstrated that combined Yoda1 and FSS treatment significantly elevated both p‐Akt and phospho‐Cx43 (Ser373) protein levels in senescent MLO‐Y4 osteocytes (Figure S13F,G). These data support a key role for PI3K‐Akt signaling in Yoda1‐mediated mechanoresponsiveness in aged bone. Interestingly, enrichment analyses also identified loading‐induced upregulation of PI3K‐Akt signaling pathway enrichment in aged tibiae treated with Yoda1 + Gap19 (Figure S14A,B), suggesting that PI3K‐Akt signaling acts as the downstream pathway of Piezo1, independently of Cx43 HC activity.

We went on to examine whether PI3K inhibition abolishes Yoda1‐induced mechanoresponsiveness in aged male mice. The PI3K inhibitor LY294002 was co‐administered intraperitoneally with Yoda1 during the 2‐week loading regimen (Figure 6H). All mice showed good health throughout the loading experiment at this dosage (Figure S3E,F). As expected, LY294002 significantly suppressed the load‐induced increase in osteocytic EB uptake that was otherwise elevated in mice treated with Yoda1 alone (Figure 6I,J). Furthermore, μCT analysis revealed that LY294002 abolished the cortical anabolic response to tibial loading observed in aged mice treated with Yoda1 alone (Figure 6K,L). Notably, LY294002 did not significantly affect EB uptake and cortical structural parameters in nonloaded tibiae (Figure 6I–L). In summary, these findings clearly indicate that activation of Piezo1 with Yoda1 in osteocytes enhances mechanical anabolic responses in aged bone by triggering PI3K‐Akt signaling to drive Cx43 phosphorylation and HC opening. This leads to increased PGE_2_ release and subsequent SOST suppression in osteocytes, resulting in unphosphorylated β‐catenin stabilization and enhanced osteoblastic bone formation on the endosteal surface (Figure 6M).

## 3 Discussion

Against the backdrop of a globally aging population, age‐related osteoporosis has emerged as an increasingly urgent medical challenge. A key factor in its pathogenesis is the age‐associated decline in anabolic sensitivity to mechanical stimulation, which disrupts the balance between bone formation and resorption (Cunningham et al. 2023). In this study, we demonstrated that the bone anabolic responses mediated by axial cyclic compression loading are compromised in aged male mice, a phenomenon associated with a reduced Cx43 HC function in osteocytes. Furthermore, chemical activation of Piezo1 using the agonist Yoda1 restored the anabolic response to mechanical loading in aged male bone. Mechanistically, the restoration of mechanoresponsiveness by Yoda1 was attributed to its interaction with Cx43, thereby potentiating Cx43 HC activity via PI3K‐Akt signaling, which stimulated PGE_2_ release and suppressed SOST expression. This mechanism was validated by the fact that the Cx43 HC inhibitor Gap19 abolished the load‐induced anabolic responses in Yoda1‐treated aged male mice.

We herein utilized 19‐month‐old male mice, a model corresponding to 60–65‐year‐old humans (Flurkey et al. 2007), to investigate the adaptive response of the aged skeleton to tibial cyclic loading. As observed in humans, aged male mice exhibited a significant reduction in bone mass and bone formation capacity compared to their mature counterparts (Bilezikian et al. 1999). Using an established unilateral hindlimb loading model (Zhang et al. 2025; Zhao et al. 2024), we confirmed the efficacy of our loading protocol in mature (16‐week‐old) mice, which exhibited robust improvements in bone architecture and elevated bone formation on both periosteal and endosteal surfaces. In contrast, aged male mice subjected to the same mechanical strains showed no significant improvements in cortical bone or most trabecular parameters, a finding that aligns with our previous report (Zhao et al. 2024). Consequently, this study provides strong evidence to identify the attenuated adaptive response to mechanical loading in aged skeletons.

The mechanosensitive ion channel Piezo1 is well established as a critical mediator of bone mechanoresponsiveness. Studies in young mature mice have demonstrated that conditional deletion of Piezo1 in osteoblast lineage cells impairs the skeletal response to mechanical loading (Hendrickx et al. 2021; Li et al. 2019), whereas its activation by the agonist Yoda1 increases bone mass and upregulates bone formation‐related mechanosensitive genes (Li et al. 2019; Wasi et al. 2024). Given that Piezo1 expression declines with age in both human (Huang et al. 2025; Sun et al. 2019) and mouse (Li et al. 2023) skeletons, the age‐related attenuation of bone mechanoresponsiveness may be linked to reduced Piezo1 function. In this study, we found that activating Piezo1 with Yoda1 rescued the cortical anabolic response to 2 weeks of tibial cyclic loading in aged male mice. This effect was achieved by augmenting bone formation and suppressing bone resorption on the endosteal surface, a finding further supported by gene enrichment analysis. Our results are consistent with recent reports demonstrating additive effects between Piezo1 activation and mechanical loading. One study revealed that a 2‐week regimen combining loading and the Piezo1 agonist Yoda2 enhanced cortical bone parameters in 22‐month‐old female mice (Meslier et al. 2025). Similar results were likewise observed in middle‐aged female mice (50 weeks old) following dual treatment with Yoda1 and moderate loading (Wasi et al. 2024). It is noteworthy that 2‐week Yoda1 treatment alone failed to enhance cortical bone mass in our aged male mice. In contrast, Meslier et al. reported that Yoda2 administered at a comparable dosage and frequency significantly improved cortical bone parameters in 22‐month‐old female mice (Meslier et al. 2025). A similar trend was also observed in our 19‐month‐old female mice treated with Yoda1. One plausible reason is that bone metabolism in female mice is highly estrogen‐dependent. Estrogen deficiency significantly downregulates Piezo1 expression in postmenopausal women with osteoporosis (Huang et al. 2025). Similarly, OVX‐induced estrogen deficiency leads to decreased Piezo1 levels and pronounced bone loss in female mice, a phenomenon that is reversible by activation of Piezo1 with Yoda1 (Hu et al. 2023; Yang et al. 2024). In vivo, bone tissue cells are known to mediate local estrogen biosynthesis via aromatase (Sasano et al. 1997), forming a bone‐specific estrogen microenvironment independent of systemic circulation (Simpson et al. 2000). Activation of Piezo1 may upregulate aromatase and enhance estrogen receptor α nuclear translocation to regulate the anabolism in aged female bone (Yang et al. 2024). In contrast, Piezo1 signaling in aged males is estrogen‐independent and more complex; activating Piezo1 alone fails to effectively improve bone mass. Intriguingly, unlike its effect on cortical bone, Yoda1 did not significantly influence the trabecular bone response to mechanical loading in our aged male mice. Mechanical loading alone enhanced trabecular thickness to a similar extent in aged male mice regardless of Yoda1 treatment, which is consistent with previous studies (Meakin et al. 2014, 2015). It is likely that trabecular bone with softer and more deformable mechanical nature can experiences higher local tissue strains than cortical bone under mechanical loading (Bayraktar et al. 2004), which may promote more robust osteocyte activation and anabolic signaling. In addition, the possible chronic joint damage in aged mice may complicate the findings in the trabecular bone region (Rubin et al. 2002). Notably, periosteal bone remodeling remained unresponsive to mechanical loading even with Yoda1 treatment in aged male mice. This observation aligns with the established understanding that mechanical stimulation contributes minimally to periosteal bone formation in aged animals. Previous studies have demonstrated that age‐related cortical bone loss occurs predominantly at the endocortical surface (Kim et al. 2020), and the anabolic response in aged skeletons is largely restricted to the endosteal rather than the periosteal surface (Birkhold et al. 2016, 2017; Zhao et al. 2024). This could be attributed to the age‐related decline in periosteal activity and osteogenic responsiveness, in contrast to the better‐preserved osteogenic potential of the endosteum (Birkhold et al. 2016; Li et al. 2023).

Although Yoda1 is systemically distributed, we propose that osteocytes, as the principal mechanosensors within bone, may play a key role in mediating the Yoda1‐contributed anabolic response to mechanical loading. Indeed, Yoda1 treatment alone did not alter bone formation or bone resorption on the cortical surface in aged male mice, suggesting no direct effect on osteoblasts or osteoclasts. This is further supported by the observation that osteoclast‐specific deletion of Piezo1 does not affect bone mass (Wang et al. 2020), indicating that Piezo1 is dispensable for osteoclast function and effectively ruling out a direct effect of Yoda1 on osteoclasts. As expected, our in vitro experiments showed that conditioned medium from Yoda1 + FSS‐treated osteocytes both promoted osteoblastic differentiation and suppressed osteoclastogenesis. Moreover, we observed altered expression of osteocyte‐derived anabolic factors in the loaded tibiae from Yoda1‐treated aged male mice. In addition, the anabolic response to mechanical stimulation was blunted in aged osteocytes lacking Yoda1 treatment, a finding robustly demonstrated both in vivo and in vitro. Consistent with our findings, previous studies have demonstrated a critical role for osteocytic Piezo1 in mechanotransduction. Mechanical stimulation upregulates Piezo1 expression and activity in osteocytes (Li et al. 2019; Lin et al. 2022), but not in osteoblasts (Hendrickx et al. 2021). Piezo1 activation by Yoda1 promotes the nuclear translocation of YAP/TAZ, leading to increased Wnt1 transcription and protein expression in osteocytes (Li et al. 2019; Lin et al. 2025). Overexpression of Piezo1 in osteocytes augments the production of bone anabolic factor PGE_2_ under mechanical stimulation (Li et al. 2019). Conversely, either pharmacological inhibition with GsMTx4 (Liu et al. 2022; Sasaki et al. 2020) or genetic knockdown of Piezo1 (Sasaki et al. 2020) in osteocytes abolishes the mechanical induction of anabolic gene expression. It should be noted, however, that a recent study utilizing osteocyte‐specific Cx43‐deficient mice driven by the S*ost* promoter reported that Piezo1 in osteocytes is not required for the response to mechanical loading (Li et al. 2025). Since SOST is expressed only in a subset of osteocytes and is downregulated by mechanical stimulation in young mice, it is plausible that newly formed osteocytes are not efficiently targeted by the *Sost*‐Cre transgene during mechanical loading (Fu et al. 2023).

An important question concerns how anabolic factors are released by osteocytes in response to mechanical loading. With aging, the degeneration of the osteocyte network hinders the diffusion of key signaling molecules. In this study, however, we did not detect significant structural alterations in the lacunar‐canalicular network, ruling out major changes in osteocyte connectivity as a contributing factor. Notably, communication between osteocytes and their extracellular environment represents another critical mechanism for mechanoresponsiveness. Previous studies have demonstrated that Piezo1 activation by mechanical loading triggers the opening of Cx43 HCs in osteocytes in mature mice (Zeng et al. 2022). Here, we confirmed this loading‐induced Piezo1‐Cx43 interaction and subsequent Cx43 HCs opening are impaired in senescent osteocytes. We further established Piezo1 as an upstream regulator of Cx43 HC function. In aged osteocytes, Yoda1‐induced activation of Piezo1 promoted its interaction with Cx43 on the plasma membrane under mechanical loading, leading to the opening of Cx43 HCs. Interestingly, although Yoda1 has been reported to directly stimulate Cx43 HC opening in normal osteocytes (Zeng et al. 2022), we observed that Yoda1 alone did not significantly alter Cx43‐Piezo1 co‐localization or HC activity in senescent osteocytes. We propose that multiple synergistic mechanisms underlie this context‐dependent effect. Yoda1 can reduce the mechanical threshold required to activate Piezo1 channel (Botello‐Smith et al. 2019), enabling its activation even under mild loading in aged osteocytes with reduced Piezo1 expression. In addition, mechanical stimulation directly alters the spatial conformation of Piezo1, rendering it more susceptible to activation by Yoda1 (Lin et al. 2019). Moreover, mechanical stimulation may facilitate the proper membrane assembly of Cx43 (Batra, Riquelme, Burra, and Jiang 2014) and increase its likelihood of interaction with Piezo1. Furthermore, given that aging elevates oxidative stress and impairs Piezo1 function, appropriate mechanical stimulation can activate the antioxidant system in osteocytes to counteract this impairment (Guo et al. 2025). Together, these observations indicated that Yoda1 treatment promotes the opening of Cx43 HCs under mechanical stimulation in senescent osteocytes via a certain synergistic effect.

We have previously shown that tibial loading induces the opening of Cx43 HCs in osteocytes, which in turn mediates the release of the anabolic factor PGE_2_ to promote bone formation and inhibit bone resorption on the endosteal surface (Zhao, Hua, et al. 2022; Zhao, Riquelme, et al. 2022). However, this loading‐induced PGE_2_ production is known to decline with age (Chermside‐Scabbo et al. 2020; Zhao et al. 2024). In the present study, we confirmed this age‐related decline in loading‐induced PGE_2_ and COX‐2 expression in osteocytes. Crucially, co‐treatment with Yoda1 and loading reversed this deficit, demonstrating a synergistic restoration that neither condition produced alone. PGE_2_ released through osteocytic Cx43 HCs has been shown to suppress the expression of SOST, a catabolic factor that inhibits osteogenesis and promotes osteoclastogenesis in mature mice (Zhao, Hua, et al. 2022; Zhao, Riquelme, et al. 2022). Consistently, herein we observed no change in SOST expression in vehicle‐treated aged male mice subjected to mechanical loading. In contrast, administration of Yoda1 in aged mice led to a marked decrease in SOST expression in response to mechanical loading. This was accompanied by reduced RANKL expression in osteocytes and increased β‐catenin levels in osteoblasts. Osteocytic β‐catenin also acts in a critical role in the skeletal anabolic response to mechanical loading. Previous studies have demonstrated that targeted deletion of β‐catenin in osteocytes abolishes loading‐induced bone formation in adult mice (Lara‐Castillo et al. 2015). Mechanical stimulation rapidly activates β‐catenin and promotes its nuclear translocation in osteocytes via a PGE_2_‐dependent mechanism (Kamel et al. 2010; Lara‐Castillo et al. 2015). Additionally, nuclear β‐catenin accumulation facilitates the Cx43 HC assembly (Xia et al. 2010). In line with these findings, we observed elevated unphosphorylated β‐catenin and Cx43 protein levels in osteocytes of Yoda1‐treated aged male mice following tibial loading. Importantly, the functional involvement of Cx43 HCs in Yoda1‐mediated mechanoresponsiveness was further confirmed using Gap19, a specific Cx43 HC inhibitor that effectively blocks HC activity both in vitro and in vivo. Remarkably, Gap19 treatment abolished the anabolic responses on the cortical endosteal surface in aged male mice, primarily due to impaired PGE_2_‐mediated suppression of SOST expression. Conversely, Cx43‐overexpressing senescent osteocytes exhibited enhanced anabolic response to mechanical stimulation, along with increased HC opening, underscoring the functional importance of Cx43 modulation. Together, these results indicate that Yoda1‐induced activation of Piezo1 enhances the anabolic response of aged bone to mechanical loading. This effect is primarily achieved by increasing the opening of Cx43 HCs, which promotes PGE_2_ release and then inhibits SOST and enhances β‐catenin expression in osteocytes, ultimately leading to enhanced endosteal bone formation while inhibiting bone resorption on the endosteal surface.

Previous in vitro studies have established the critical role of the PI3K‐Akt pathway in regulating Cx43 HC opening (Batra et al. 2012; Batra, Riquelme, Burra, Kar, et al. 2014; Riquelme et al. 2021; Sasaki et al. 2020; Zhao et al. 2023). Enhanced Akt phosphorylation leads to conformational change in the extended extracellular domain of integrin α5β1 (Batra et al. 2012; Batra, Riquelme, Burra, Kar, et al. 2014), and subsequently to the opening of the HC (Riquelme et al. 2021). Here, our in vitro and in vivo studies consistently demonstrate that Piezo1 activation enhances its interaction with Cx43 in aged osteocytes subjected to mechanical stimulation. This results in elevated levels of phosphorylated (active) Akt, which in turn promotes Akt‐mediated phosphorylation of Cx43 at Serine 373. Consequently, the interaction between Cx43 and integrin α5 is strengthened, thereby facilitating the opening of Cx43 HCs. It is worth noting that even when Cx43 HCs are blocked by Gap19, mechanical loading still upregulates the PI3K‐Akt pathway in Yoda1‐treated aged male mice, indicating that PI3K‐Akt signaling acts as a direct downstream pathway of Piezo1 under mechanical stimulation, and its activation is not affected by whether Cx43 HCs are open. Furthermore, the PI3K inhibitor LY294002 even completely prevented the Yoda1‐mediated HC activity in response to tibial loading in aged cortical bone, along with an impediment of anabolic response to mechanical loading in cortical bone. Thus, our results reveal a role for the Piezo1‐Akt pathway in mediating both HC opening and the anabolic response to mechanical loading.

There are several limitations of this study. First, Piezo1 and Gap19 may act not only on osteocytes but also on other cell types in bone; nevertheless, our drug treatments did not induce obvious phenotypic changes in osteoblasts or osteoclasts in aged bone. Second, while etoposide‐induced senescence in MLO‐Y4 cells recapitulates several key characteristics of naturally aged osteocytes, such as elevated SA‐β‐Gal activity and compromised mechanosensitivity, aged osteocytes in tibiae exist within a complex bone microenvironment characterized by dynamic crosstalk between diverse bone cell populations. This physiological complexity is not reproduced in the etoposide‐induced senescence model, which also fails to mimic age‐related structural and compositional alterations in the osteocytic lacuno‐canalicular network and extracellular matrix. Third, estrogen deficiency has been reported to downregulate Piezo1 expression and Cx43 HC function in bone tissue (Huang et al. 2025; Ma et al. 2019; Yang et al. 2024). Whether estrogen deficiency impairs Cx43 HC function by reducing Piezo1 expression remains to be further explored.

## 4 Conclusion

This study unveils a crucial relationship between Piezo1 and Cx43 HCs in the mechanoresponsiveness of aged bone. Activation of Piezo1 with Yoda1 restores the anabolic response to mechanical loading in aged tibiae, an effect primarily mediated by the Piezo1‐Cx43 interaction that potentiates hemichannel activity. The opening of Cx43 HCs promotes PGE_2_ release and suppresses SOST expression, thereby stimulating bone formation on the endosteal surface. Our findings underscore the therapeutic potential of combining low‐magnitude mechanical loading with Piezo1 agonism to reduce the loading dosage required for effective bone formation, thereby improving patient compliance and minimizing the risk of overuse injury. Future studies should explore the safety, optimal dosing regimen, and long‐term skeletal effects of systemic or local Piezo1 activation, as well as its potential off‐target effects on other mechanosensitive tissues.

## 5 Methods

### 5.1 Animals

All mice were maintained under standard pathogen‐free conditions until reaching their target ages, respectively. To minimize potential confounding effects from frequent aggressive encounters, male mice were singly housed. All described animal protocols were reviewed and approved by the Institutional Animal Care and Use Committee of Northwest University.

### 5.2 Experimental Administration

One hour prior to mechanical loading, randomly allocated 19‐month‐old WT mice received intraperitoneal injections of Yoda1 (HY‐18723, MedChemExpress) or vehicle for five consecutive days per week over a 2‐week period (days 1–5 and 8–12). Mice were euthanized on day 15 for subsequent analysis. Yoda1 (SML1558, MedChemExpress) was initially dissolved in dimethyl sulfoxide (DMSO) then diluted in 5% ethanol for injection. The compound was administered intraperitoneally at a dosage of 5 μmol/kg body weight, according to previously established protocols (Li et al. 2019). To inhibit Cx43 HCs, TAT‐Gap19 (HY‐18723, MedChemExpress), a potent and selective Cx43 HC blocker, was dissolved in filter‐sterilized saline and co‐administered with Yoda1 at a dose of 5 mg/kg body weight, as previously described (Quan et al. 2022). To inhibit the PI3K/Akt pathway, the PI3K inhibitor LY294002 (HY‐18723, MedChemExpress) was dissolved in filter‐sterilized saline containing 10% DMSO and administered either alone or together with Yoda1 at 8 mg/kg body weight (Gong et al. 2023). Vehicle groups received the same amounts of solvents: 5% ethanol for Yoda1, sterilized saline for Yoda1 + Gap19, and 10% DMSO for Yoda1 + LY294002.

### 5.3 In Vivo Tibial Loading

As previously described (Zhao, Hua, et al. 2022; Zhao, Riquelme, et al. 2022; Zhao et al. 2024), the left tibia was stabilized in a custom loading fixture under a 0.5 N static preload (Figure S1A,B) and then subjected to cyclic loading at 2 Hz for 600 cycles. The peak load was determined from established load‐strain calibration curves (Meakin et al. 2014; Zhao, Riquelme, et al. 2022) to achieve a peak periosteal strain of 1200 με at the cortical mid‐diaphysis in both mature and aged mice. This strain magnitude has been shown in our previous work to elicit an anabolic response at the same site in mature mice (Zhao, Riquelme, et al. 2022). The right tibiae served as contralateral, non‐loaded controls.

### 5.4 Micro‐Computed Tomography

Bone microstructure was evaluated by a high‐resolution micro‐computed tomography (μCT) system (VivaCT 80, Scanco Medical) after 2‐week tibial loading. Two volumes of interest (VOIs) were analyzed: one in the metaphyseal trabecular bone and another in the cortical region (Figure S1D). For trabecular bone, the VOI was positioned at a point 0.45 mm (45 slices) distal to the proximal growth plate and extending distally over a 1 mm (100 slices) span. For cortical bone, the VOI was positioned at the mid‐diaphysis, corresponding to 50% of the total bone length from the proximal end, and consisted of a 0.5 mm (50 slices) segment, as described previously (Li et al. 2025; Zhao, Riquelme, et al. 2022).

### 5.5 Dynamic Bone Histomorphometry

Mice received intraperitoneal injections of calcein (C0875, Sigma‐Aldrich) 1 day before the commencement of tibial loading and a second injection 3 days prior to euthanasia, following established protocols (Zhao, Hua, et al. 2022; Zhao, Riquelme, et al. 2022; Zhao et al. 2024). The tibiae were embedded in methyl methacrylate, and transverse sections of the mid‐diaphyseal region were prepared. Fluorescent labeling was visualized with a motorized fluorescence microscope (Observer 3, ZEISS).

### 5.6 In Vivo HC Dye Uptake

Osteocytic HC activity in tibiae was evaluated in situ according to established protocols (Acosta et al. 2024; Zhao, Hua, et al. 2022; Zhao, Riquelme, et al. 2022; Zhao et al. 2024). Briefly, Evans Blue (E2129, Sigma‐Aldrich) dye (20 mg/mL) was injected intravenously via the tail vein. Mice then underwent 10 min of mechanical loading, followed by a 40‐min rest period. Afterwards, the anesthetized animals were transcardially perfused to achieve in situ fixation of tibial osteocytes. Frozen sagittal sections were prepared to assess EB fluorescence intensity within the mid‐diaphyseal region.

### 5.7 PGE_2_ Measurement

The tibial diaphysis was carefully dissected to remove bone marrow and soft tissues and collected 4 h after completion of the 5‐day mechanical loading. Following homogenization (SWE‐FP PLUS, Servicebio), PGE_2_ concentrations in the homogenates were determined by an ELISA kit (514,010, Cayman Chemical) and normalized to total protein content, as measured by a BCA assay kit (P0010S, Beyotime).

### 5.8 Histology

Following a 2‐week mechanical loading regimen, tibiae were decalcified, sagittally embedded in paraffin, and sectioned into 5‐μm‐thick longitudinal slices. Tartrate‐resistant acid phosphatase (TRAP) staining was performed to evaluate osteoclast activity. Multinucleated TRAP‐positive osteoclasts (containing ≥ 3 nuclei) were quantified along endosteal surfaces. Osteoblasts were identified by toluidine blue staining as previously described (Zhao, Hua, et al. 2022; Zhao, Riquelme, et al. 2022; Zhao et al. 2024). Hematoxylin and eosin (H&E) staining was employed to examine potential drug toxicity in internal organs. The osteocyte lacuno‐canalicular network was visualized and quantified using Ploton silver staining (Zhao, Hua, et al. 2022).

### 5.9 Immunohistochemistry

Immunohistochemical staining was performed using the VECTASTAIN ABC kit (PK‐4001/PK‐4005, Vector Laboratories) with DAB substrate (SK‐4100, Vector Laboratories). After deparaffinization and antigen retrieval, tissue sections were incubated with the following primary antibodies: COX‐2 (27308‐1‐AP, Proteintech; 1:200), Osteocalcin (23418‐1‐AP, Proteintech; 1:100), SOST (AF1589, R&D Systems; 1:100), Cx43 (C6219, Sigma‐Aldrich; 1:50), p‐Akt (4060, Cell Signaling Technology; 1:50), and unphosphorylated β‐catenin (51067‐2‐AP, Proteintech; 1:200).

### 5.10 Cell Culture

The MLO‐Y4 osteocytic cell line was generously provided by Dr. Lynda Bonewald. The culture medium consisted of α‐minimal essential medium (α‐MEM) with 2.5% fetal bovine serum (FBS) and 2.5% calf serum (CS) supplemented with 1% penicillin/streptomycin. To establish a cell line stably overexpressing *Gja1*, MLO‐Y4 cells were transduced with a lentiviral vector containing the *Gja1* sequence (Genecarer Bio., China). Cells transduced with an empty lentiviral vector served as the negative control (Figure S9A). The efficiency of *Gja1* overexpression was confirmed by RT‐qPCR (Figure S9B). The osteoblast‐like cell line MC3T3‐E1 and the osteoclast precursor line RAW264.7 were maintained in α‐MEM supplemented with 10% FBS including 1% penicillin/streptomycin, while RAW264.7 cells were cultured in α‐MEM containing 5% FBS and 1% penicillin/streptomycin. All cells were maintained in a humidified incubator at 37°C with 5% CO_2_.

### 5.11 Senescent Cell Viability

To induce cellular senescence, MLO‐Y4 cells were seeded in 96‐well plates at a density of 1 × 10^4^ per well and pre‐cultured for 12 h. The cells were then treated with etoposide, a topoisomerase inhibitor that induces DNA damage and cellular senescence (Petrova et al. 2016) (HY‐13629, MedChemExpress), at concentrations of 0, 5, 10, 20, and 40 μM for 24 and 48 h. Following treatment, cell viability was quantified using a Cell Counting kit‐8 (CCK‐8) (C0041, Beyotime).

### 5.12 SA‐β‐Gal Staining

SA‐β‐galactosidase (SA‐β‐Gal) activity was detected using a commercial Senescence β‐Galactosidase Staining Kit (C0602, Beyotime) according to the manufacturer's instructions. Senescent cells, identified by green staining, were visualized under a bright‐field microscope (CKX53, Olympus).

### 5.13 Fluid Shear Stress Stimulation

MLO‐Y4 cells were grown at a low density on Type I collagen‐coated glass slides to ensure that most cells were not in physical contact. The cells were then preincubated with 5 μM etoposide for 24 h to induce senescence (Moiseeva et al. 2023; Petrova et al. 2016). After pre‐treatment, the cells were incubated in fresh culture medium containing one of the following for 1 h: vehicle (0.1% DMSO), 30 μM Yoda1, and 30 μM Yoda1 + 20 μM Gap19 (Kim et al. 2019; Sun et al. 2019). The CCK‐8 assay confirmed that 30 μM Yoda1 had no effects on cell viability (Figure S4B). The slides were then transferred to a parallel‐plate flow chamber as previously described (Xu et al. 2014). The 1‐h exposure to 16 dyn/cm^2^ FSS at 1 Hz was selected for subsequent assays, as it elicited a marked upregulation of key mechanoresponsive genes (Figure S4C).

### 5.14 Conditioned Medium Treatment

For osteogenic differentiation, MC3T3‐E1 cells were cultured in a 1:1 mixture of conditioned medium and fresh culture medium, supplemented with 50 μg/mL β‐glycerophosphate, 50 μg/mL ascorbic acid, and 10 μM dexamethasone. After 5 days of differentiation, osteoblasts were visualized using a BCIP/NBT Alkaline Phosphatase Color Development Kit (C3206, Beyotime). For osteoclastogenesis, RAW264.7 cells were cultured in a 1:1 mixture of conditioned medium and fresh RAW264.7 culture medium, containing 50 ng/mL recombinant RANKL (Tnfsf11‐6551 M, Creative BioMart). After 5 days of induction, osteoclasts were stained using a tartrate‐resistant acid phosphatase (TRAP) staining kit (G1492, Solarbio).

### 5.15 In Vitro Dye Uptake

Following FSS exposure, the cell‐covered slides were processed for dye uptake analysis. Cells were incubated with 25 μM ethidium bromide (EtBr) in recording medium, which consisted of HCO₃^−^‐free α‐MEM buffered with 10 mM HEPES. After incubation, the cells were fixed with 1% paraformaldehyde (158127, Sigma‐Aldrich) for 10 min.

### 5.16 Immunofluorescence

On completion of the FSS, the cells were fixed and incubated simultaneously with primary antibodies against Cx43 (C6219, Sigma‐Aldrich; 1:400) and Piezo1 (MA5‐32876, Invitrogen; 1:100), then incubated with Cy3‐conjugated goat anti‐rabbit secondary antibody (A10520, Invitrogen; 1:300) and FITC‐conjugated goat anti‐mouse secondary antibody (F‐2761, Invitrogen; 1:100). Nuclei were counterstained with DAPI, and protein co‐localization was visualized using a confocal laser microscope (SP5, Leica).

### 5.17 Proximity Ligation Assay (PLA)

Cells grown on round microscope coverglasses were blocked and incubated with the mixture of mouse anti‐Cx43 (3D8A5, Thermo Scientific; 1:100) and rabbit anti‐Piezo1 (15939‐1‐AP, Proteintech; 1:800), or the mixture of mouse anti‐Cx43 and rabbit anti‐integrin α5 (10569‐1‐AP, Proteintech; 1:1200) at 4°C overnight. Cells were washed and processed for PLA used the NaveniFlex Cell Red kit (60,025, Naveni), according to the manufacturer's published protocol.

### 5.18 Western Blotting

Total protein was extracted from cells using lysis buffer with 1% protease inhibitor on ice. Proteins on nitrocellulose membranes were detected with the following primary antibodies: anti‐phospho‐Cx43 (Ser373, 12594, Signalway Antibody; 1:800), anti‐phospho‐Akt (Ser473, #4060, Cell Signaling; 1:1000), and anti‐Akt (10176‐2‐AP, Proteintech; 1:1000). GAPDH (81640‐5‐RR, Proteintech; 1:5000) was used as the internal reference.

### 5.19 RNA Extraction and Quantitative Real‐Time PCR

Total RNA was extracted using TRIzol Reagent (15596018CN, Invitrogen). RNA purity and concentration were assessed using the NanoDrop 2000 spectrophotometer (Thermo Scientific). Complementary DNA (cDNA) was synthesized with a high‐capacity cDNA reverse transcription kit (AG11728, Accurate Biotechnology). Quantitative real‐time PCR (qRT‐PCR) was performed using SYBR Green (1725124, Bio‐Rad) on a CFX96 system. *Gapdh* was used as the reference gene for mRNA normalization, and relative gene expression was calculated using the 2^−ΔΔCt^ method. All primer sequences are listed in Table S1.

### 5.20 RNA‐Seq

Total RNA was extracted from marrow‐flushed tibiae as described above, and RNA integrity was confirmed using an Agilent 2100 Bioanalyzer (Agilent Technologies). Sequencing libraries were prepared with the VAHTS Universal V6 RNA‐seq Library Prep Kit and sequenced on an Illumina NovaSeq 6000 platform (OE Biotech Co. Ltd.) to generate 150 bp paired‐end reads. Raw sequencing reads in FASTQ format were quality‐controlled and trimmed using fastp software to obtain high‐quality clean reads. Approximately 49 million clean reads per sample were retained for subsequent analysis. These reads were aligned to the reference genome using HISAT2. The expression level of each gene was quantified as FPKM, and read counts were generated using HTSeq‐count. Differential gene expression analysis was performed with DESeq2, applying a significance threshold of a *q* value < 0.05 for DEGs.

### 5.21 Phosphorylation Site Prediction

The full‐length amino acid sequence of mouse Cx43 (UniProtKB/Swiss‐Prot: CXA1_MOUSE, P23242) was retrieved for in silico phosphorylation analysis. Akt‐targeted phosphorylation sites were predicted using Scansite 4.0 and validated with GPS 6.0, with Ser373 identified as the top high‐confidence candidate. The 3D structure of Cx43 was modeled with AlphaFold2 to localize Ser373, and a sequence logo of the Akt consensus motif was generated via WebLogo 3.0 to confirm motif conservation.

### 5.22 Statistics

Statistical analysis was conducted using GraphPad Prism Version 9 software. Variance homogeneity was evaluated using the Levene test, and normal distribution was determined by the Shapiro–Wilk test. The paired 2‐tailed Student's *t*‐test was used to compare the contralateral and loaded tibiae within each mouse. The unpaired 2‐tailed Student's *t*‐test was employed for comparisons between independent groups in the in vitro experiments. One‐way ANOVA with Tukey test was used for multiple group comparisons for only one condition. Two‐way ANOVA with Tukey test was used for multiple group comparisons for two conditions. All values at *p* < 0.05 were considered to be significant. All experimental data were shown as mean ± standard deviation (SD).

## Author Contributions

D.Z., J.F., Q.G., J.W., and H.X. conceived the idea and designed the experiments. D.Z., J.F., H.L., S.L., J.W., M.S., A.Z., and Y.Z. performed the experiments. D.Z., J.F., H.L., and Q.G. analyzed the data and designed the figures. D.Z. wrote the first draft of the manuscript and H.X. revised the manuscript. D.Z. wrote the definitive manuscript. D.Z., D.W., J.W., and H.X. provided the resources. Both D.Z. and J.F. made equal contributions. D.Z. wrote the first draft and is listed first. All authors edited and commented on previous versions of the manuscript and approved the final version.

## Funding

This work was supported by the National Natural Science Foundation of China (Nos. 32301088 and 81772409) and the Natural Science Foundation of Shaanxi Provincial Department of Education (No. 23JK0688).

## Conflicts of Interest

The authors declare no conflicts of interest.

## Supporting information


**Figure S1:** Experimental setup for tibia axial compressive loading.
**Figure S2:** Etoposide induces senescence in MLO‐Y4 osteocytic cells.
**Figure S3:** Histopathological evaluation of major visceral organs.
**Figure S4:** Experimental setup for in vitro fluid flow stimulation.
**Figure S5:** Effects of Yoda1 and Gap19 on the gene expression of osteocyte‐related cytokines in senescent MLO‐Y4 osteocytic cells under FSS.
**Figure S6:** Yoda1 and loading mitigated the loss of cortical and trabecular bone in aged female mice.
**Figure S7:** In situ proximity ligation assay (PLA) for protein interactions between Piezo1 and Cx43, as well as between Cx43 and integrin α5 in FSS‐stimulated senescent MLO‐Y4 cells treated with Yoda1.
**Figure S8:** Effects of conditioned medium from FSS‐stimulated senescent MLO‐Y4 cells treated with Yoda1 or Yoda1 + Gap19 on osteoclast and osteoblast differentiation.
**Figure S9:** Cx43 overexpression enhances mechanosensitivity in senescent MLO‐Y4 osteocytic cells under fluid flow stimulation.
**Figure S10:** Blocking Cx43 HCs abolishes the load‐induced PGE_2_ release and SOST reduction in osteocytes observed in Yoda1‐treated aged male mice.
**Figure S11:** Effects of Yoda1 and Gap19 treatment on canalicular density under mechanical loading.
**Figure S12:** RNA‐Seq of tibiae reveals the change in osteoblast activity in Yoda1‐treated aged male mice under mechanical loading.
**Figure S13:** The potential Akt phosphorylation sites on the Cx43 predicted by in silico analysis.
**Figure S14:** RNA‐Seq of tibiae reveals that PI3K‐Akt signaling acts as a direct downstream pathway of Piezo1 gating under mechanical stimulation, independently of Cx43 hemichnnels.

**Table S1:** Primers used for qRT‐PCR.

## Data Availability

Sequencing data are available at the Sequence Read Archive, under accession no. PRJNA1355301. The data supporting the findings of this study are available from the corresponding author upon request.
