# Supplementary material for: Piezo1 Activation Rescues Anabolic Response to Mechanical Loading in Aged Bone via Connexin 43 Hemichannels
Source: Aging Cell. 2026 Aug 21;25(9):e70687. doi: 10.1111/acel.70687 (PMC13498897; doi:10.1111/acel.70687)
Supplement: Supplementary file 1 [file ACEL-25-e70687-s001.docx]

**Figure S1. Experimental setup for tibia axial compressive loading.** (**A**) Custom cyclic loading device for applying uniaxial compression to the mouse tibia in vivo. (**B**) Positioning of the left tibia within the loading device and the direction of the applied force. (**C**) Schematic of the overall experimental timeline. The PGE_2_ was measured on day 5, while bone structure, bone histomorphometry, immunohistochemistry (IHC), and RNA-Seq were evaluated on day 15.

**Figure S2. Etoposide induces senescence in MLO-Y4 osteocytic cells.** (**A** and **B**) Viability of MLO-Y4 cells treated with etoposide at concentrations of 0μM, 5μM, 10μM, 20μM, 40μM for 24 hours (**A**) and 48 hours (**B**). n = 4-6 per group. (**C**) Representative images of SA–β-gal staining in MLO-Y4 cells treated with etoposide at concentrations of 5, 10, 20, 40μM for 24 hours. Scale bar: 100 μm. (**D**) Proportion of SA–β-gal-positive MLO-Y4 cells normalized to total cell number. n = 6 per group. (**E**) mRNA expression of senescent marker genes in MLO-Y4 cells treated with 5μM etoposide for 24 hours, including p16, p21 and p53. n = 3 per group. Data are expressed as mean ± SD. (**A**, **B**, and **D**) ^***^P<0.001 and ^****^P<0.0001 by one-way ANOVA with Tukey test for multiple group comparisons. (**E**) ^*^P<0.05 and ^***^P<0.001 by the unpaired Student’s t test.

**Figure S3. Histopathological evaluation of major visceral organs.** (**A**) Weekly body weights of aged male mice treated with Yoda1 or Yoda1+Gap19. n = 10-14 per group. (**B**) Representative H&E-stained sections of major organs (heart, liver, spleen, lung, and kidney) from aged male mice treated with Yoda1 or Yoda1+Gap19. (**C**) Wet weight of major organs (heart, liver, spleen, lung, and kidney) normalized to body weight in aged male mice treated with Yoda1 or Yoda1+Gap19. n=12-19 per group. (**D**) Weekly body weights of aged female mice treated with Yoda1. (**E**) Weekly body weights of aged male mice treated with LY294002 or Yoda1+LY294002. n=10-14 per group. (**F**) Wet weight of major organs (heart, liver, spleen, lung, and kidney) normalized to body weight in aged male mice treated with LY294002 or Yoda1+LY294002. Data are expressed as mean ± SD. Statistical analysis was performed using two-way ANOVA with Tukey test (**A**, **C**-**E**) or unpaired Student’s t test (**F**). Yd1, Yoda1; G19, Gap19; LY, LY294002.

**Figure S4. Experimental setup for in vitro fluid flow stimulation.** (**A**) Schematic of the protocol for collecting conditioned medium from senescent MLO-Y4 cells treated with Yoda1 or Yoda1+Gap19 under fluid shear stress (FSS). The conditioned mediumz and MLO-Y4 cells were collected for further detection. (**B**) Viability of senescent MLO-Y4 cells treated with Yoda1 at concentrations of 1μM, 5μM, 10μM, 20μM, 30μM, 40μM and 50μM for 1 hour. n = 3 per group. (**C**) Time-course analysis of mechanoresponsive gene expression in normal MLO-Y4 cells subjected to 16 dyn/cm² FSS at the indicated time points. Data are expressed as mean ± SD. (**C**) ^*^P<0.05, ^**^P<0.01, and ^***^P<0.001 by one-way ANOVA with Tukey test for multiple group comparisons.

**Figure S5. Effects of Yoda1 and Gap19 on the gene expression of osteocyte-related cytokines in senescent MLO-Y4 osteocytic cells under FSS.** (**A**) Gene expression of *Tnfsf11* in senescent MLO-Y4 cells treated with Yoda1 in response to FSS stimulation. (**B**) Gene expression of *Tnfrsf11b*, *Ptgs2*, and *Ctnnb1* in senescent MLO-Y4 cells treated with Yoda1 with or without Gap19 under FSS stimulation. Data are expressed as mean ± SD. (**A** and **B**) ^#^P<0.05 and ^##^P<0.01 by unpaired Student’s t test. (**A** and **B**) ^*^P<0.05, ^**^P<0.01, and ^***^P<0.001 by two-way ANOVA with Tukey test for differences among groups. Eto, etoposide; Yd1, Yoda1; G19, Gap19.

**Figure S6.** **Yoda1 and loading mitigated the loss of cortical and trabecular bone in aged female mice.** All 19-month-old female mice were administered 5 μmol/kg Yoda1 or vehicle for five consecutive days per week over a 2-week period. (**A** and **B**) Representative μCT 3D images and quantitative analysis of metaphyseal trabecular bone in vehicle- and Yoda1-treated aged female mice. n = 5 per group. (**C** and **D**) Representative 3D images and quantitative analysis of mid-diaphyseal cortical bone in vehicle- and Yoda1-treated aged female mice. n = 5 per group. (**E and F**) Calcein double labeling of the mid-diaphysis in vehicle- and Yoda1-treated aged female mice. Scale bar, 100 μm. n = 5 per group. Data are expressed as mean ± SD. (**C**, **E**, **G**) ^#^P<0.05 and ^##^P<0.01 by the paired Student’s t test. (**C**, **E**, **G**, **H**) ^*^P<0.05, ^**^P<0.01, and ^***^P<0.001 by two-way ANOVA with Tukey test.

**Figure S7. *In situ* proximity ligation assay (PLA) for protein interactions between Piezo1 and Cx43, as well as between Cx43 and integrin α5 in FSS-stimulated senescent MLO-Y4 cells treated with Yoda1.** (**A** and **B**) Representative images of the interaction of piezo1and Cx43 visualized by PLA and quantification of PLA puncta per cell. Each red spot represents for a single interaction. Scale bar, 10 μm. n = 4 per group. (**C** and **D**) Representative PLA images showing the interaction between Cx43 and integrin α5 and quantification of PLA puncta per cell. Each red spot represents for a single interaction. Scale bar, 10 μm. n = 4 per group. Data are expressed as mean ± SD. (B and D) ^###^P<0.001 by unpaired Student’s t test. (B and D) ^***^P<0.001 by two-way ANOVA with Tukey test for differences among groups. Eto, etoposide; Yd1, Yoda1.

**Figure S8. Effects of conditioned medium from FSS-stimulated senescent MLO-Y4 cells treated with Yoda1 or Yoda1+Gap19 on osteoclast and osteoblast differentiation.** (**A**) ALP staining of MC3T3-E1 cells cultured in conditioned medium from senescent MLO-Y4 cells treated with Yoda1 or Yoda1+Gap19 under FSS. (**B**) The corresponding quantification of ALP-positive area (%). (**C**) Representative TRAP staining of RAW264.7 cells cultured in conditioned medium from senescent MLO-Y4 cells treated with Yoda1 or Yoda1+Gap19 under FSS. (**D**) The corresponding quantification of the average number of nuclei and cell area per mature osteoclast. Data are expressed as mean ± SD. (**B** and **D**) ^#^P<0.05, ^##^P<0.01, and ^###^P<0.001 by unpaired Student’s t test. (**B** and **D**) ^*^P<0.05 and ^**^P<0.01 by two-way ANOVA with Tukey test for differences among groups. Yd1, Yoda1; G19, Gap19.

**Figure S9. Cx43 overexpression enhances mechanosensitivity in senescent MLO-Y4 osteocytic cells under fluid flow stimulation.** (**A**) Lentiviral vector map for Gja1 overexpression. (**B**) qRT-PCR confirmation of *Gja1* overexpression in MLO-Y4 cells. n = 4 per group. (**C** and **D**) The quantification (**C**) and representative images of EtBr dye uptake (**D**) in normal and Cx43-overexpressing senescent MLO-Y4 cells in response to FSS stimulation. n = 3 per group. (**E**) Expression of *Ptgs2* and *Ctnnb1* in normal and Cx43-overexpressing senescent MLO-Y4 cells under FSS. n = 3-4 per group. (**F** and **G**) Representative ALP staining of MC3T3-E1 cells cultured in conditioned medium from normal and Cx43-overexpressing senescent MLO-Y4 cells in response to FSS stimulation, and quantification of ALP-positive area (%). n = 4 per group. Data are expressed as mean ± SD. (**B**) ^*^P<0.05 by unpaired Student’s t test. (**C**, **E**, and **G**) ^#^P<0.05 by unpaired Student’s t test. (**C**, **E**, and **G**) Two-way ANOVA with Tukey test was used for differences among groups. OE, overexpression, NC, negative control.

**Figure S10. Blocking Cx43 HCs abolishes the load-induced PGE_2_ release and SOST reduction in osteocytes observed in Yoda1-treated aged male mice.** (**A**) The PGE_2_ levels in the tibial diaphysis after 5 days of mechanical loading in Yoda1- and Yoda1+Gap19-treated aged male mice. n = 5 per group. (**B** and **C**) Representative COX-2 immunohistostaining and quantification of COX-2-positive osteocytes in mid-diaphyseal cortical bone from Yoda1- and Yoda1+Gap19-treated aged male mice. Scale bar: 40 μm. n = 5 per group. (**D**-**I**) Immunohistochemical staining and corresponding quantification of SOST (**D** and **E**), β-catenin (**F** and **G**), and Cx43 (**H** and **I**) protein expression in osteocytes (brown; black arrows) from mid-diaphyseal cortical bone of Yoda1- and Yoda1+Gap19-treated aged male mice. Scale bar, 40 μm. n = 5 per group. (**J** and **K**) β-catenin immunohistochemical staining for labeling osteoblasts (brown, black arrows) on endosteal surfaces and the corresponding statistical result. Scale bar, 40 μm. n = 5 per group. Data are expressed as mean ± SD. (**A**, **C**, **E**, **F**, **I**, and **J**) ^#^P<0.05 and ^##^P<0.01 by the paired Student’s t. (**A**, **C**, **E**, **F**, **I**, and **J**) ^*^P<0.05, ^**^P<0.01, and ^***^P<0.001 by two-way ANOVA with Tukey test. Yd1, Yoda1; G19, Gap19.

**Figure S11. Effects of Yoda1 and Gap19 treatment on canalicular density under mechanical loading.** (**A** and **B**) Representative images of Ploton silver staining showing the osteocyte lacunar-canalicular network in tibiae from aged male mice treated with vehicle or Yoda1 after cyclic compressive loading (**A**), and the corresponding quantification of the lacunar-canalicular area normalized to total bone area (**B**). Scale bar, 20 μm. n = 5 per group. (**C** and **D**) Representative images of Ploton silver staining showing osteocyte lacunar-canalicular network in tibiae from aged male mice treated with Yoda1 or Yoda1+Gap19 following cyclic compressive loading (**C**), and the corresponding quantification of lacunar-canalicular normalized to total bone area (**D**). Scale bar, 20 μm. n = 5 per group. Data are expressed as mean ± SD. Statistical analysis was performed using paired Student’s t test for loaded versus contralateral tibiae, and two-way ANOVA with Tukey test was used for differences among groups. Yd1, Yoda1; G19, Gap19.

**Figure S12. RNA-Seq of tibiae reveals the change in osteoblast activity in Yoda1-treated aged male mice under mechanical loading.** (**A**) Number of loading-induced differentially expressed genes (DEGs) in Yoda1- and vehicle-treated aged male mice. (**B**) Gene ontology (GO) enrichment analysis of loading-regulated genes in Yoda1- and vehicle-treated aged male mice. (**C**) Heatmap of DEGs related to osteoblast differentiation based on the GO analysis. (**D**) Gene set enrichment analysis (GSEA) plot showing significant enrichment of osteoblast differentiation-related signaling events in loaded versus contralateral tibiae of Yoda1-treated aged male mice. (**E**) KEGG pathway analysis showing the top 20 enriched KEGG pathways. (**F**) Wikipathway analysis showing the top 10 enriched pathways between the loaded and contralateral tibiae in vehicle-treated mice.

**Figure S13. The potential Akt phosphorylation sites on the Cx43** **predicted by in silico analysis.** (**A**)Tabulated results showing the location and sequence of high-scoring motif sites, along with their percentile rank and surface accessibility (SA) when compared with the total number of all possible motif sites within the vertebrate subset of SWISS-PROT. (B) Protein plot analysis of mouse Cx43 (via UniProtKB/Swiss-Prot) identifies the predicted Akt phosphorylation site at serine 373 (S373, top panel). The bottom panel shows the surface accessibility profile. (**C**) Graphical representation of the percentile rank of mouse Cx43 Ser373, a predicted most high-confidence Akt phosphorylation site, relative to the distribution of Akt kinase motif scores across all vertebrate proteins in the UniProtKB/Swiss-Prot database. (**D**) The three-dimensional structure of mouse Cx43 demonstrates that Ser373, the most probable Akt phosphorylation site, is localized to the C-terminal domain of Cx43. (**E**) Sequence logo illustrating the conserved Akt kinase phosphorylation motif. (**F**) Western blotting analysis of pAkt and pCx43 (Ser373) proteins from senescent MLO-Y4 cells treated with Yoda1 in response to FSS. (**G**) Quantification of p-Akt/Akt and p-Cx43/GAPDH. n=3 per group. Data are expressed as mean ± SD. ^*^P<0.05 by unpaired Student’s t test.

**Figure S14. RNA-Seq of tibiae reveals that PI3K-Akt signaling acts as a direct downstream pathway of Piezo1 gating under** **mechanical stimulation , independently of Cx43 hemichnnels.** RNA-seq analysis was performed to compare loaded versus nonloaded contralateral tibiae from aged male mice treated with Yoda1+Gap19. (**A**) Volcano plots showing the genes upregulated and downregulated in loaded versus contralateral tibiae from Yoda1+Gap19-treated aged male mice. Blue and red dots represent significantly regulated genes (|log_2_ fold change| >1, p-value < 0.05). **(B)** Wikipathway analysis showing the top enriched pathways between the loaded and contralateral tibiae in Yoda1+Gap19-treated aged male mice.
